# Transcellular propagation of fibrillar α-synuclein from enteroendocrine to neuronal cells requires cell-to-cell contact and is Rab35-dependent

**DOI:** 10.1038/s41598-022-08076-5

**Published:** 2022-03-09

**Authors:** Paulla Vieira Rodrigues, João Vitor Pereira de Godoy, Beatriz Pelegrini Bosque, Dionísio Pedro Amorim Neto, Katiane Tostes, Soledad Palameta, Sheila Garcia-Rosa, Celisa Caldana Costa Tonoli, Hernandes Faustino de Carvalho, Matheus de Castro Fonseca

**Affiliations:** 1 Brazilian Center for Research in Energy and Materials (CNPEM), Brazilian Biosciences National Laboratory (LNBio), 10000 Giuseppe Maximo Scolfaro St., Campinas, São Paulo, 13083-100 Brazil; 2 Department of Structural and Functional Biology, State University of Campinas, Campinas, São Paulo, Brazil; 3 Present Address: Laboratory of Sarkis Mazmanian, Division of Biology and Biological Engineering, California Institute of Technology, 1200 E. California Boulevard, Pasadena, CA USA

**Keywords:** Cell signalling, Cellular imaging, Mechanisms of disease

## Abstract

Parkinson’s disease (PD) is a neurodegenerative condition featured by motor dysfunction, death of midbrain dopaminergic neurons and accumulation of α-synuclein (αSyn) aggregates. Growing evidence suggests that PD diagnosis happens late in the disease progression and that the pathology may originate much earlier in the enteric nervous system (ENS) before advancing to the brain, via autonomic fibers. It was recently described that a specific cell type from the gut epithelium named enteroendocrine cells (EECs) possess many neuron-like properties including αSyn expression. By facing the gut lumen and being directly connected with αSyn-containing enteric neurons in a synaptic manner, EECs form a neural circuit between the gastrointestinal tract and the ENS, thereby being a possible key player in the outcome of PD in the gut. We have characterized the progression and the cellular mechanisms involved in αSyn pre-formed fibrils (PFFs) transfer from EECs to neuronal cells. We show that brain organoids efficiently internalize αSyn PFF seeds which triggers the formation of larger intracellular inclusions. In addition, in the enteroendocrine cell line STC-1 and in the neuronal cell line SH-SY5Y, αSyn PFFs induced intracellular calcium (Ca^2+^) oscillations on an extracellular Ca^2+^ source-dependent manner and triggered αSyn fibrils internalization by endocytosis. We characterized the spread of αSyn PFFs from enteroendocrine to neuronal cells and showed that this process is dependent on physical cell-to-cell contact and on Rab35 GTPase. Lastly, inhibition of Rab35 increases the clearance of αSyn fibrils by redirecting them to the lysosomal compartment. Therefore, our results reveal mechanisms that contribute to the understanding of how seeded αSyn fibrils promote the progression of αSyn pathology from EECs to neuronal cells shifting the focus of PD etiology to the ENS.

## Introduction

Neurodegenerative diseases characterized by aberrant aggregates of insoluble amyloid α-synuclein (αSyn) fibrils are collectively referred to as synucleinopathies and constitute the second most common form of neurodegenerative dementias (Lewy body dementia and Parkinson's disease with dementia)^1^. αSyn is a 140 amino acid protein broadly expressed in the brain, mostly localized in the cytosol and at presynaptic terminals in association with synaptic vesicles. However, its exact function is not completely elucidated^2,3^. Misfolded αSyn may aggregate in specific neuronal cell populations forming intraneuronal inclusions called Lewy Bodies (LB) leading to distinct phenotypes. For example, intraneural inclusions in midbrain dopaminergic cells are associated with motor dysfunction in PD^4^. Nevertheless, growing evidence indicates that synucleinopathy diagnosis happens late in disease progression and that pathology may originate much earlier in the gastrointestinal (GI) tract before advancing to the brain. This has its base on the fact that fibrillar forms of αSyn, the main component of LBs, can be transmitted from cell to cell in a prion-like manner^5–9^. In addition, biopsies of GI tissue from patients with PD have found αSyn accumulation in the stomach, duodenum and colon in cells of the enteric nervous system (ENS)^10,11^.

Heterologously expressed αSyn can be aggregated in vitro to form fibrils similar in structure to those found in vivo^12^. These pre-formed fibrils (PFF) can spread in a prion-like manner both in in vitro neuronal cultures and in vivo when injected into the mouse brain. In addition, injection of αSyn fibrils into the gut wall of mice converts endogenous αSyn to pathologic species that spread to the brain through vagal fibers that innervate the gut, causing behavioral dysfunction^13,14^.

It is noteworthy that although αSyn aggregation was observed in the ENS, these neurons and enteric glial cells are not the only cell populations that express αSyn. It was recently described that a specific cell type from the gut epithelium named enteroendocrine cells (EECs) possess many neuron-like properties including αSyn expression^15^. EECs face the gut lumen and connect directly with αSyn-containing enteric nerves in a synaptic manner forming a neural circuit between the gut and the ENS. Since these cells are directly influenced by the gut lumen microenvironment, it has been suggested that the process of αSyn misfolding might start in EECs^16^ and αSyn aggregates could propagate from the gut epithelium to the ENS and then to the central nervous system (CNS).

However, the progression and mechanisms of αSyn transfer from EECs to neuronal cells have not been characterized. In this work we show that αSyn PFFs can be efficiently internalized by iPSCs-derived brain organoids and initiate an aggregation process. In experiments performed with the enteroendocrine cell line STC-1 and the neuronal cell line SH-SY5Y, αSyn PFFs induced intracellular calcium (Ca^2+^) oscillations on an extracellular Ca^2+^ source-dependent manner and triggered αSyn fibrils internalization by endocytosis. In addition, the transcellular propagation of αSyn PFFs from enteroendocrine to neuronal cells is dependent on physical cell-to-cell contact and on Rab35 GTPase. Lastly, inhibition of Rab35 increases the clearance of αSyn fibrils by redirecting them to the lysosomal compartment.

Together, our results reveal mechanisms that contribute to the understanding of how seeded αSyn fibrils promote the progression of αSyn pathology from EECs to neuronal cells.

## Methods

### Cell lines

STC-1 (CRL-3254) and SH-SY5Y cell lines were obtained from the America Type Culture Collection (ATCC). STC-1 and SH-SY5Y cell lines were cultured in DMEM and DMEM/F12 (Gibco), respectively, supplemented with 10% fetal bovine serum (FBS), 1% penicillin/streptomycin antibiotics (PSA) and incubated at 37 ºC with 5% CO2: 95% air.

### Expression and purification of recombinant human αSyn

The expression and purification of human WT αSyn (M1-140) has been described elsewhere^17–19^. Briefly, the HsαSyn fusion construct in pET21b vector was expressed in *E.coli* BL21 (DE3) bacteria. The supernatant was purified with anion exchange chromatography using a HiScreen Q HP (GE HealthCare). Fractions containing the protein were then submitted to size-exclusion chromatography using a Superdex 75 16/60 prepgrad column (GE Healthcare). The purified eluted protein was dialyzed against 50 mM Tris–HCl pH 7.5 and 50 mM NaCl, overnight at 4ºC. Final concentration was estimated by the BCA assay according to the manufacturer’s instructions (Thermo Fisher Scientific).

### Preparation of αSyn fibrils

The expression and purification of human (Hs) WT αSyn was performed as previously described^20^. For fibril formation, αSyn was incubated in buffer A (50 mM Tris–HCl, pH 7.5, 150 mM KCl) at 37 °C under continuous shaking in an Eppendorf Thermomixer set at 600 r.p.m. Fibrillar αSyn was centrifuged twice at 15,000 g for 10 min, resuspended twice in PBS and labeled with Alexa Fluor 488, 555 or 633 Protein Labeling Kit (Thermo Fisher Scientific) following the manufacturer’s instructions using a protein:dye ratio of 1:2. The labeling reactions were arrested by addition of 1 mM Tris pH 7.5. The unreacted fluorophore was removed by a final cycle of two centrifugations at 15,000 g for 10 min and resuspensions of the pelleted fibrils in PBS. The nature of PFFs forms was assessed by Circular Dichroism (CD), Dynamic Light Scattering (DLS) and Western Blotting.

### Circular Dichroism (CD)

Proteins at 15 µM in buffer (50 mM Tris–HCl, 150 mM KCl, pH 7.5) were evaluated using a Jasco J-810 spectropolarimeter equipped with a Peltier system PFD 425S with temperature control. CD spectra were obtained at 10 ºC or 37 °C using a 0.1-mm path length with cell response time of 4 s, scanning speed of 100 nm/min and at 1-nm intervals, over the wavelength range from 195 to 260 nm. For all the samples analyzed, each data point was produced by averaging ten accumulations.

### Dynamic light scattering (DLS)

For Dynamic Light Scattering analysis, αSyn monomers or PFFs (15 µM) in buffer (50 mM Tris–HCl, 150 mM KCl, pH 7.5) were centrifuged (13,000 rpm, 10 min at 4 °C) and submitted to DLS measurements. Data were recorded in a quartz cell with a scattering angle of 90°at 25 °C on a Malvern Zetasizer Nano ZS90 (Malvern Instruments, Worcestershire, UK) equipped with 632.8 nm He–Ne laser. Measurements of each sample refers to 15–100 acquisitions of 10 s, with an automatic attenuator (Attenuation 11). Z-average radius (Z-ave), intensity size distribution, and the polydispersity index (PdI) were obtained from the autocorrelation function using the “Protein Analysis mode” for the protein sample. Data were collected and analyzed with Malvern ZetaSizer Software version 7.11.

### Generation of iPSCs and iPSC-derived brain organoids

Erythroblasts from three normal controls (Wild Type—WT) were infected with Sendai virus (Life Technologies, USA), accordingly to manufacturer's instructions. Four days post-infection erythroblasts were plated on the iMEF feeders and cultured using hESC medium. After 3–4 weeks, iPSC clones were manually picked and were further propagated clonally on feeders. The brain organoid differentiation method used was similar to Lancaster et al.^21^, with minor modifications^21^. On day 0 of organoid culture, iPSCs were dissociated by accutase (Millipore) treatment to generate single cells. In total, 9000 cells were plated in each well of an ultra-low-binding 96-well plate (Corning) in human mTeSR1 with 50 µM Rho-associated protein kinase (ROCK) inhibitor (Peprotech). On day 2, the media was changed to hES media with low concentration basic fibroblast growth factor (4 ng/ml) and 50 µM Rho-associated protein kinase (ROCK) inhibitor (Peprotech). Embryoid bodies were fed every other day for 6 days then transferred to low-adhesion 24-well plates (Corning) in neural induction media containing Dulbecco's modified eagle medium (DMEM)/F12, 1:100 N2 supplement (Invitrogen), Glutamax (Invitrogen), minimum essential media-nonessential amino acids (MEM-NEAA—Invitrogen) and 1 µg/ml heparin (Sigma-Aldrich). These began forming neuroepithelial tissues, which were fed every other day for 5 days. On day 11, tissues were transferred to droplets of growth reduced Matrigel (Corning) by pipetting into 25 µl cold Matrigel on a sheet of Parafilm. These droplets were allowed to gel at 37 °C and were subsequently removed from the Parafilm, transferred (no more than 20) to low-adhesion 100 mm plates (Corning) and grown in differentiation media containing a 1:1 mixture of DMEM/F12 and Neurobasal media containing 1:200 N2 supplement (Invitrogen), 1:100 B27 supplement without vitamin A (Invitrogen), 50 µM beta-mercaptoethanol (Gibco), 2.5 µg/ml insulin (Sigma-Aldrich), 1% Glutamax and 0, 5% MEM-NEAA and 1% penicillin and streptomycin (Invitrogen). After 4 days of stationary growth, the tissue droplets were transferred to an orbital shaker under agitation of 85 RPM containing differentiation media as above except B27 supplement with vitamin A (Invitrogen) was used. Organoids were used after 60 days of differentiation.

### Cell transfection

STC-1 and SH-SY5Y cell lines were transfected with full-length human GFP-tagged αSyn or pcDNA3.1-GFP-2a-αSyn-RFP using FuGene HD (Promega) according to manufacturer's instructions. Cells were used 48 h after transfection. pcDNA3.1-GFP-2a-αSyn-RFP^22,23^ was kindly donated by Dr. Eleanna Kara (University College London, London, UK).

### αSyn PFFs internalization assay in cell lines

STC-1 or SH-SHSY cells were treated with 1 μM of Alexa-Fluor-tagged human recombinant αSyn PFFs, unless otherwise stated. Internalization was evaluated after 24 or 48 h by immunofluorescence and confocal microscopy after washing with PBS and fixing the cells with 4% PFA at room temperature for 20 min. The percentage of Alexa-Fluor-positive cells was analyzed using the ImageJ software.

### Cytosolic calcium measurements

For cytosolic calcium measurements, cells were loaded with the Ca^2+^ indicator Fluo-4/AM (Thermo Fisher Scientific) for 15 min at 37ºC, placed onto the stage of a Leica SP8 Confocal System and continuously perfused with HEPES buffer solution or HEPES Ca^2+^-free buffer (HEPES buffer solution: 142.2 mM NaCl, 5.4 mM KCl, 1.0 mM NaH_2_PO4, 10 mM HEPES, 5.6 mM dextrose, 0.8 mM MgSO_4_ and 1 mM CaCl_2/_ HEPES Ca^2+^-free buffer: 142.2 mM NaCl, 5.4 mM KCl, 1.0 mM NaH_2_PO4, 10 mM HEPES, 5.6 mM dextrose, 0.8 mM MgSO_4_ and 1 mM EGTA). 1 µM αSyn PFFs was used to trigger Ca^2+^ release. Data are expressed as fluorescence/baseline fluorescence × 100% of the average values of samples from 3 biological replicates (at least 25 cells were individually analyzed). The images were obtained using a Leica SP8 confocal microscope, using a × 63 objective lens, 1.4 NA, excitation at 488 nm and emission at 505–525 nm for both dyes.

### Monitoring endocytosis with FM1-43

STC-1 or SH-SHSY cells were incubated for 10 min in HEPES buffer solution (142.2 mM NaCl, 5.4 mM KCl, 1.0 mM NaH_2_PO_4_, 10 mM HEPES, 5.6 mM dextrose, 0.8 mM MgSO_4_ and 1 mM CaCl_2_) containing either 4 µM FM1-43 or fixable FM1-43 analog (FM1-43fx). After, cells were placed onto the stage of a Leica SP8 Confocal System and continuously perfused with HEPES buffer solution also containing the dye and stimulated with 1 µM PFFs. Cells were imaged for 90 min using a Leica SP8 confocal microscope, using a × 63 objective lens, 1.4 NA, excitation at 488 nm and emission at 505–525 nm. Data are expressed as fluorescence/baseline fluorescence × 100% of the average values of samples from 3 biological replicates (at least 25 cells were individually analyzed).

### Western immunoblot

Cell lysates were obtained by treating cells with RIPA buffer supplemented with inhibitors of proteases and phosphatases followed by centrifugation at 10,000 rpm for 10 min at 4 °C. 35 µg proteins were separated on 10% SDS–polyacrylamide or non-denaturing polyacrylamide gels. Proteins were transferred to nitrocellulose membranes (Millipore) and blocked in 3% (w/v) skimmed milk in Tris-buffered saline/0.1% Tween 20 (TBS-T). After, membranes were incubated over-night at 4ºC in primary antibody [(rabbit anti-alpha synuclein (Abcam), mouse β-actin (Santa Cruz) or rabbit anti-Rab35 (Abcam)] diluted in blocking solution. Membranes were washed with PBS and incubated with secondary antibody for 1 h at room temperature. The membranes were washed again in TBS-T and the signal visualized with ECL reagent (Millipore).

### Immunofluorescence

Cell cultures grown on coverslips were fixed with 4% PFA followed by permeabilization with 0.1% Triton X-100 and for 15 min at room temperature (RT) and then, blocked with 5% donkey serum for 1 h at RT. Primary antibody (rabbit anti-Rab35, 1:100; Abcam; rabbit anti-pser129 αSyn, 1:150; Abcam) diluted in antibody diluent (1% donkey serum + 0.1% Triton X-100) was incubated overnight at 4 °C. Secondary antibody (Goat-anti-rabbit Alexa 488, 1:500) was incubated for 1 h at RT. Cells were washed three times with PBS + 0.1% Tween-20. Coverslips were mounted on glass slides using VectaShield with DAPI mounting medium (Vector laboratories).

For whole brain organoids immunofluorescence, after being treated with 1 µM Alexa-633-tagged αSyn PFFs for 24 or 48 h, tissues were fixed in 4% PFA for 20 min at 4 °C followed by washing in PBS three times for 10 min before embedding in 4% low melting agarose (Sigma-Aldrich) in 1X PBS. Tissue sections of 50 µm were obtained using a vibratome (Leica VT1000 S Vibratome, Leica Microsystems, Wetzlar, Germany). For immunofluorescence, sections were blocked and permeabilized in 0.3% Triton X-100 and 4% FBS in PBS for 2 h at RT. Sections were then incubated with phalloidin Alexa-488 (Thermo Fisher, USA). Sections were washed three times with PBS + 0.1% Tween-20 and mounted on slides using VectaShield with DAPI mounting medium (Vector laboratories).

### Lysosome staining with Lysotracker

To investigate whether αSyn PFFs would co-localize with lysosomes, cells treated with Alexa-tagged αSyn fibrils were incubated with LysoTracker Green or Deep Red (Life Technologies) for 30 min at 1:100 dilution. Cells were then fixed with 4% PFA for 20 min, washed with PBS and mounted on slides using VectaShield with DAPI mounting medium (Vector laboratories). Co-localization analysis were performed in blind-coded slides.

### STC-1 – SH-SY5Y co-culture

Acceptor cells were plated on coverslips at the bottom of 6-well plate at a density that would allow sub-confluency to be reached after 10 h. The donor cells were then treated with αSyn fibrils as described above for 24 h. The day after, donor cells were detached and mixed with acceptor cells at the ratio of 1:1 and plated. Cells were kept in co-culture for up to 7 days. In the case of siRNA-based silencing or transfection, cells were silenced/transfected for 24 h, then treated with PFFs on the next day for 24 h and kept in co-culture for the described time. Percentage of transferred PFFs were analyzed in blind-coded slides.

### Co-culture system in transwell filters

To co-culture donor and acceptor cells in conditions that allow their physical separation, acceptor cells were plated on coverslips at the bottom of 6-well plates. An equal number of donor cells containing internalized Alexa-555-tagged αSyn PFFs were plated in transwell filters with 0.4 µM-pore polyester membrane insert (Sigma-Aldrich) which were then placed within the 6-well plates. Experiments were performed so both STC-1 and SH-SY5Y could act as donor or acceptor cells. After 24,48 or 72-h co-culture, the filter was removed, the acceptor cells on the coverslip and donor cells on the membrane were fixed, stained with DAPI, Alexa-488 phalloidin and imaged Leica SP8 confocal microscope, using a × 63 objective lens, 1.4 N.A.

### Flow cytometry

Flow cytometry analysis were performed according to Abounit, 2016^24^. Briefly, STC-1 and SH-SY5Y cells were plated in 6-well plates at a density that would allow sub-confluency to be reached after 10 h. Then, STC-1 cells were treated with 1 µM of Alexa-Fluor-488-tagged human recombinant αSyn PFFs and SH-SY5Y cells with Alexa-Fluor-555-tagged αSyn PFFs. Fibrils were sonicated prior to internalization for 5 min at 80% amplitude with a pulse cycle of 5 s on and 2 s off. After 24 h, cells were washed, trypsinized and co-cultured for 24, 48 or 72 h. To remove plasma membrane-bound assemblies, cells were washed three times with 0.1% trypsin (Gibco) and were detached by pipetting and then passed through sterile 40 µm nylon cell strainers (BD FalconTM) in order to obtain single-cell suspensions. Cells were fixed using 4% PFA. The percentage of Alexa-488 , Alexa-555-positive cells or cells positive for both dyes at each time point were scored using Novocyte flow cytometer (ACEA).

### Silencing with siRNA

The validated Pre-designed Invitrogen Silencer ® siRNAs Silencer® (Thermo Fisher) sense and antisense oligonucleotides for mouse Rab35 (ID 176,638) and scrambled sequences were purchased. RNAiFect Reagent (Qiagen) was used to deliver each siRNA as previously described. Silenced STC-1 cells were used 48 h after siRNA treatment as indicated. In case of co-transfection with pEGFP plasmid and siRNA silencing, TransMessenger Reagent was used. The recommended protocols for co-transfection of adherent cells with siRNA and plasmid DNA using TransMessenger Reagent was followed. Only GFP-positive cells were analysed. Fluorescence intensity of PFFs and co-localization analysis in control and silenced cells were performed in blind-coded slides.

### Image analysis

The levels of co-localization, fluorescence intensity and percentage of transferred PFFs were analyzed in cells from random chosen fields using ImageJ program (NIH) in a blinded fashion. To evaluate PFFs transfer, Z-slices of 0.25 µM were manually analyzed to avoid misleading analysis. Analyses of calcium signals and vesicle endocytosis were also performed in ImageJ by selecting a ROI in the cytoplasm of individual cells and monitoring fluorescence signal from the same ROI during time-course. Analyses of western blots was performed in ImageJ or Image Lab (Bio-Rad). Regions of interest (ROIs) were drawn around bands of interest or the length of the lane and the integrated density was measured. Relative integrated densities were calculated against the average of the WT samples on each membrane and normalized to housekeeping genes.

### Statistical analysis

The statistical relevance of the bar graphs was obtained by calculating the P-value using the paired two-tailed Student’s *t*-test or One-Way ANOVA as detailed in each figure legend. Number of experiments and cells analyzed is detailed in figure legends. The bar graphs showed in the figures are presented as mean ± S.E.M. All column graphs, plots and statistical analyses were done using GraphPad Prism version 5 software.

## Results

### αSyn PFFs are efficiently internalized by iPSC-derived human brain organoids

**Figure 1:**
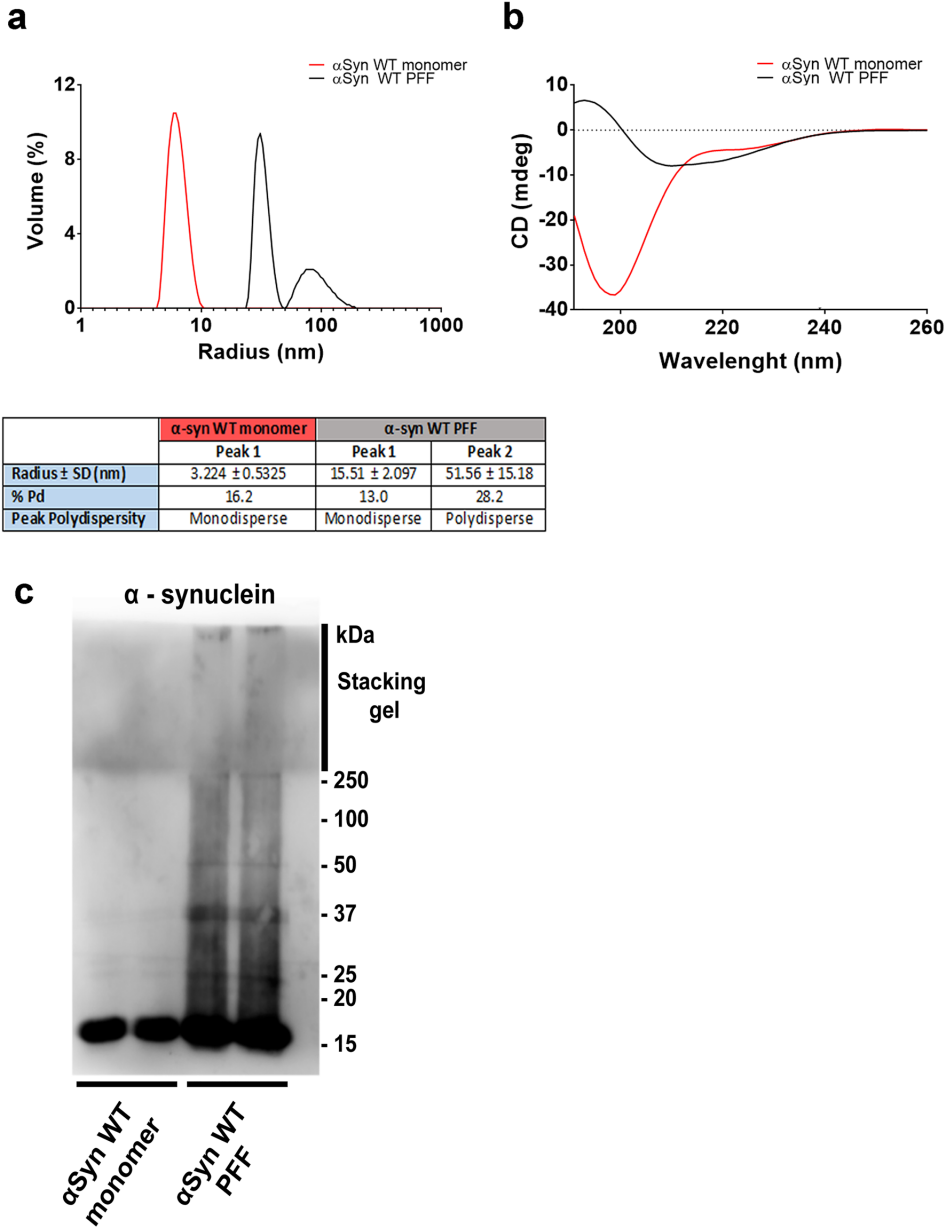
Characterization of purified αSyn monomers and fibrils. (**a**) DLS of αSyn monomers before (red line) and after PFF formation (black line). Table below shows hydrodynamic radius, percentage (%) of polydispersity and peak polydispersity for each peak represented. (**b**) CD of αSyn monomers before (red line) and after PFF formation (black line). (**c**) Immunoblotting of purified human αSyn WT before (lanes 1 and 2) and after 7 days of incubation at 37 ºC and agitation at 600 r.p.m. for fibril formation (lanes 3 and 4).

Administration of αSyn PFFs is a recognized, established model of idiopathic synucleinopathy in the CNS that closely represents human pathology^7,8^. To investigate whether αSyn PFFs could be internalized by different cell types we first expressed and purified recombinant human αSyn monomers. After, the protein was submitted to the process of PFF assembly, as previously described^20^. As shown in Fig. 1, biophysical and biochemical characterization of samples indicated successful production of αSyn PFFs, which feature higher hydrodynamic radius and polydispersity when compared to αSyn monomers (Fig. 1a). CD spectra shows that the aggregation process happens via α-helix-rich conformation (exemplified with single minima at ~ 200 nm for αSyn monomers) before converting into a β-sheet-rich structure with single minima at ~ 220 nm (Fig. 1b) leading the monomers to assemble into higher molecular mass structures (Fig. 1c). After PFF characterizing and fluorescent labeling with Alexa-Fluor fluorophore, we assessed whether these recombinant Alexa-Fluor-tagged αSyn PFFs could initiate the spread of αSyn pathology in two different systems: iPSC-derived human brain organoids and by mice duodenum. Initially, we targeted these models because brain organoids represent a more synaptically interconnected system, and the duodenum because of its dense innervation by vagal fibers, which are thought to be the major element for the gut-to-brain hypothesis of synucleinopathy etiology ^25^.

iPSC-derived human brain organoids were generated and characterized. After 60 days of differentiation, histological and morphological analysis were employed to identify structures expressing the progenitor cell marker SOX2 and mature neuron marker MAP2 (Supplementary Fig. 1). Once differentiated, brain organoids were incubated with 1 µM of Alexa-633-tagged αSyn PFFs for 24 or 48 h. We observed that immediately after 24 h-incubation period, organoids presented several Alexa-633-positive puncta within their structures (Supplementary Fig. 2a). To gain more insights, we followed the growth of intracellular Alexa-633-positive αSyn aggregates for 48 h after seeding. After 48 h of seeding, several cells developed aggregates with increases in diameter rearranged into inclusions that morphologically resemble to the bona fide human LBs (Supplementary Fig. 2b). When we quantified the mean number of Alexa-633-positive aggregates with diameter larger than 0.5 µm we observed that organoids seeded with αSyn PFFs presented a ~ 20-fold increase in number of these larger aggregates after 48 h (Supplementary Fig. 2c). This data indicate that brain organoids efficiently internalize αSyn PFF seeds which triggers the formation of larger intracellular inclusions.

### αSyn fibrils induce the aggregation of soluble αSyn after internalization in enteroendocrine cells

Several reports have identified αSyn accumulation in neurons and enteric glial cells (EGCs) of the ENS in GI biopsies from patients with PD^10,11^. This suggests that αSyn aggregation may originate in peripheral tissues and progress to the brain via autonomic fibers. It was recently reported that EECs which are part of the gut epithelium and are directly exposed to the gut lumen and its microbiome, possess many neuron-like properties, such as αSyn expression, and are synaptically connected to enteric nerves^15^. Therefore, we hypothesized that αSyn misfolding and aggregation could initiate in this cell type and then progress to the ENS.

**Figure 2:**
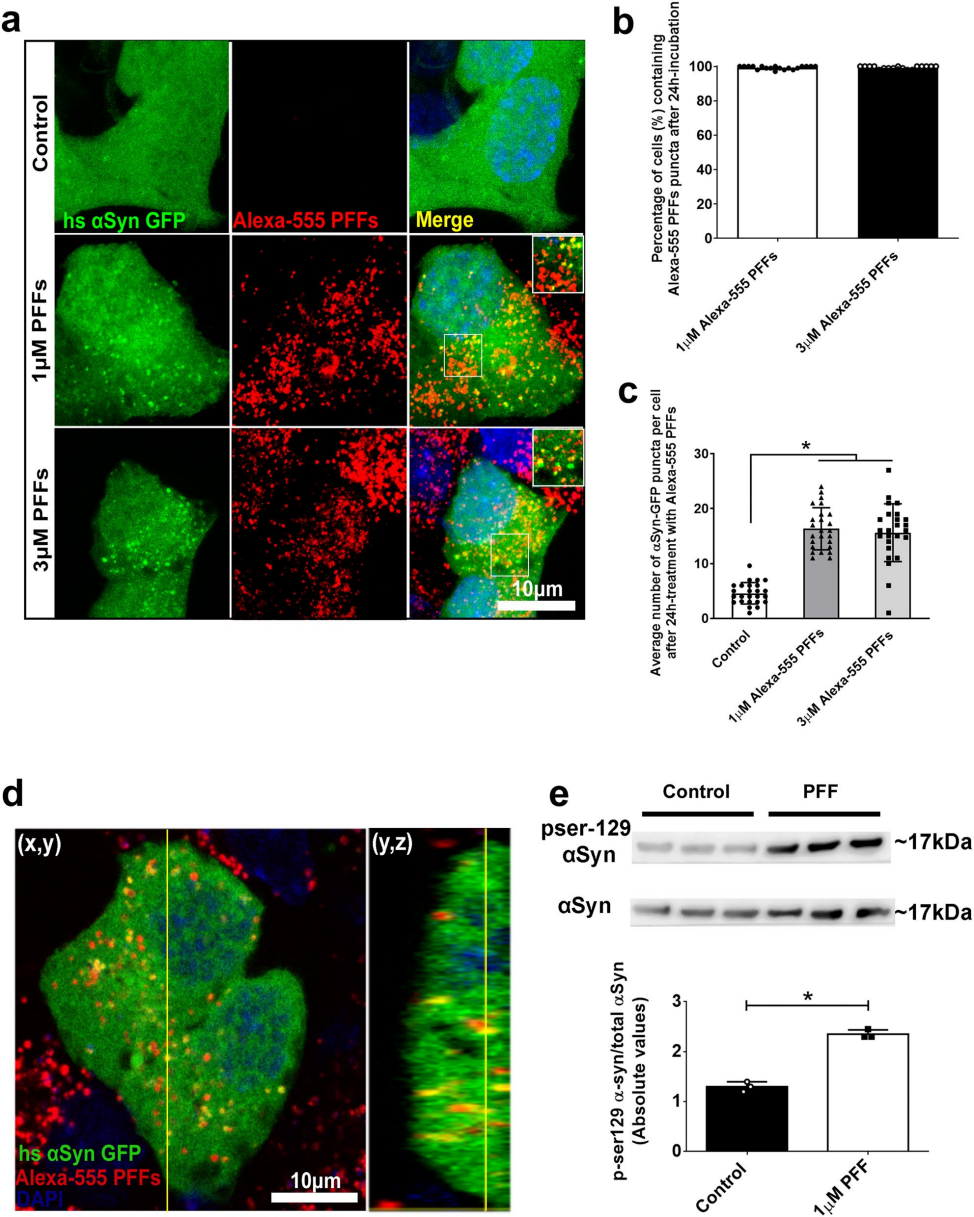
αSyn PFFs induce the aggregation of the soluble αSyn protein in enteroendocrine cells. (**a**) Representative confocal images showing GFP-αsyn-transfected cells (in green) treated with 0 (control), 1 or 3 µM of fluorescent Alexa-555 αSyn fibrils (in red) for 24 h . Inserts show αSyn PFFs co-localized with GFP- αSyn puncta. Scale bars represent 10 µm. Nuclei are stained with DAPI (blue). n = 3 independent experiments. (**b**) Quantification of the number (%) of GFP-αSyn positive cells containing Alexa-555 PFFs when cells were incubated with 1 or 3 µM of PFFs from 3 independent experiments. Data are shown as mean ± S.E.M. **c** Graph shows the mean number of GFP-αSyn puncta in transfected cells (in green) treated with 0 (control), 1 or 3 µM of fluorescent Alexa-555 αSyn fibrils (in red) for 24 h. The dots represent the mean ± S.E.M. number of 3 independent experiments in which at least 25 cells per experiment were analyzed. (**d**) High magnification image of a GFP-αSyn positive STC-1 cell treated with 1 µM of fluorescent Alexa-555 αSyn fibrils (in red) shows co-localization of GFP-αSyn positive puncta with Alexa-555-PFFs. Orthogonal slice (y,z) show that this event happens within the cell cytoplasm. Scale bar, 10 µm. (**e**) Immunoblots (upper image) of total cell lysates showing the increased phosphorylation of αSyn on Ser129 (normalized against total αSyn) in control cells or after 48 h of incubation with 1 µM αSyn PFF. Densitometric analysis shows increased phosphorylation of αSyn (pSer129) in PFF-treated group when compared to control. * *p* < 0.05 by unpaired Student’s t-test.

Although extensively shown in neurons^26,27^, a key question here is whether and how these fibrils would be internalized by the EECs, seed cytoplasmic soluble αSyn aggregation and then be transferred to neurons. To answer these questions, we worked with the enteroendocrine cell line STC-1 which is widely used as a model of native EECs^28^ due to the expression of several GI hormones and neuronal-like features, including the expression of αSyn ^15,16^. STC-1 cells were transfected with the construct pEGFP-Hs-αSyn and treated with 1 or 3 µM Alexa-555-PFFs for 24 h. While cells untreated with PFFs presented αSyn-GFP smoothly distributed throughout cell cytoplasm and nucleus, we observed that αSyn fibrils are efficiently internalized and seeded the aggregation of soluble reporter αSyn (GFP-αSyn) when exogenously added to STC-1 cells in culture (Fig. 2a,b and d). Interestingly, we did not detect any increase in the number of αSyn-GFP puncta per cell when 3 µM PFFs were applied in comparison to 1 µM (Fig. 2c). In addition, exogenous Alexa-633 PFFs co-localized with GFP-αSyn puncta (Fig. 2d). This experiment was also performed with SH-SY5Y cells and similar results were obtained (Supplementary Fig. 3). Next, we aimed to answer whether the internalization of these pathological forms of αSyn could increase the intracellular levels of phosphorylated forms of αSyn, a hallmark of PD that was shown to exacerbate the formation of αSyn inclusions^29^. Quantification by Western blotting of total cell lysates after 48 h of incubation with αSyn PFFs showed ~ twofold increase of p-Ser129-αSyn in cells treated with the αSyn PFFs when normalized against total αSyn and compared to untreated cells (Fig. 2e).

Together, these results suggest that αSyn fibrils not only recruits soluble GFP-αSyn in EECs but also increases intracellular levels of p-Ser129 αSyn.

### αSyn fibrils induce intracellular Ca^2+^ signaling and vesicle budding in STC-1 and SH-SY5Y cells

**Figure 3:**
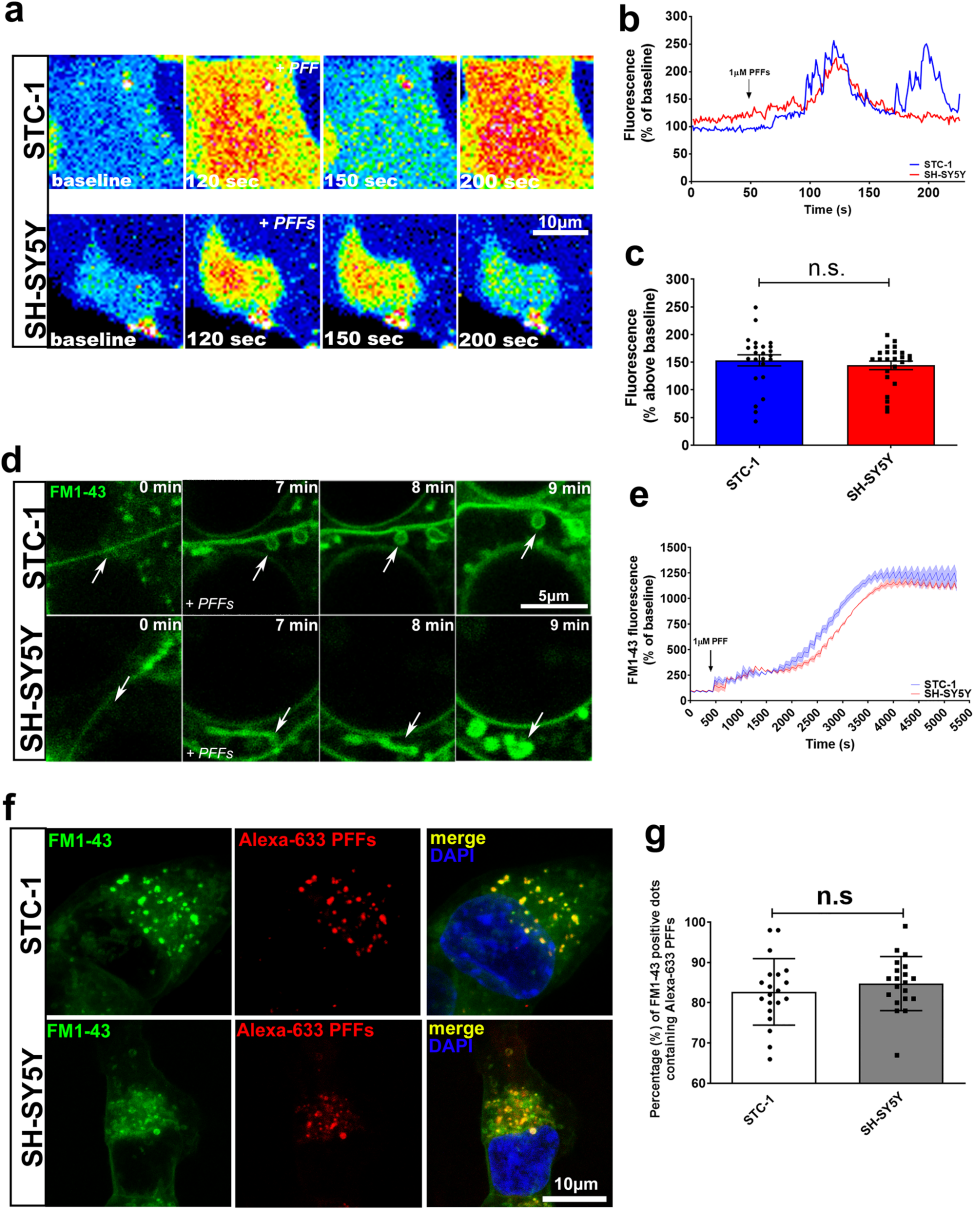
αSyn PFFs trigger intracellular Ca^2+^ and vesicle budding in STC-1 and SH-SY5Y cells. (**a**) Confocal microscopy imaging of STC-1 (upper panels) and SH-SY5Y (bottom panels) cells incubated with Fluo-4/AM (6 μM) and stimulated with 1 µM αSyn PFFs (scale bar: 10 μm). (**b**) Representative time‐course of total Ca^2+^ signal. Arrow indicates the moment when PFFs were applied. (**c**) Quantification of the peak fluorescence following stimulation with 1 µM αSyn PFFs. (Error bars indicate the media ± SEM; n = at least 25 cells for each group, *p < 0.05 by unpaired Student’s t-test). (**d**) Confocal microscopy imaging of STC-1 (upper panels) and SH-SY5Y (bottom panels) cells incubated with FM1-43 (4 μM) and stimulated with 1 µM αSyn PFFs (scale bar: 10 μm). Arrows indicate membrane regions with vesicles formation after stimulation with PFFs. (**e**) Time‐course of intracellular FM1-43 fluorescence before and after stimulation with PFFs for 5,500 s. (**f**) Confocal microscopy images of STC-1 (upper panels) and SH-SY5Y (bottom panels) cells incubated with Fluo-4/AM (6 μM) and stimulated with 1 µM αSyn PFFs-Alexa-633 for 10 min. Merged images show co-localization of FM1-43-positive vesicles with Alexa-633-positive dots (scale bar: 10 μm). (**g**) Graph shows percentage FM1-43 positive dots containing Alexa-633-positive PFFs. (Error bars indicate the media ± SEM; n = at least 20 images for each group from 3 independent experiments, *p < 0.05 by unpaired Student’s *t*-test).

It has been demonstrated that αSyn induces an increase in basal intracellular Ca^2+^ in its unfolded monomeric state as well as in its oligomeric state once applied to neurons and astrocytes^30^. However, it is still unknown whether this mechanism might play a role for PFFs internalization in EECs. To address this question, we live-imaged Fluo-4/AM-loaded STC-1 and SH-SY5Y cells during stimulation with PFFs. Interestingly, two patterns of αSyn-PFF-induced Ca^2+^ signaling were observed. In STC-1 cells, PFFs induced several Ca^2+^ spikes while in SH-SY5Y cells the kinetics of Ca^2+^ signal was featured by a large single spike also with complete recovery to baseline (Fig. 3a,b). However, no differences were observed in Ca^2+^ amplitudes occurring in response to PFF stimulation between cell lines (Fig. 3c). To determine the source of the Ca^2+^, cells were stimulated in Ca^2+‐^free medium. We observed that αSyn-PFF-induced Ca^2+^ oscillations are completely blocked when extracellular Ca^2+^ is absent strongly suggesting the role of extracellular Ca^2+^ sources for the αSyn-fibrils-induced Ca^2+^ signaling (Supplementary Fig. 4).

To demonstrate whether this Ca^2+^ transients could trigger endocytic responses to internalize αSyn PFFs in STC-1 cells, we used the vital dye FM1-43 to monitor endocytosis. FM1–43 binds membranes and during endocytosis it is internalized and trapped in vesicles, offering a measure of endocytic efficiency^31–33^. Figure 3d shows STC-1 and SH-SY5Y cells stained with FM1-43 before and during stimulation with αSyn PFFs. FM1-43 (4 µM) was present in the bathing solution in all images. Because the fluorescence of the dye is much higher once bound to membranes than when soluble in water, it selectively stains the cell surface membrane. After stimulation with αSyn PFFs, several events of vesicle budding from the plasma membrane were observed in both cell types (Fig. 3d; Supplementary Movies 1 and 2). When we monitored and plotted the average FM1-43 fluorescence intensity within the cells during image collection we observed a massive increase in fluorescence signal due to FM1-43 internalization (Fig. 3e). To confirm that αSyn-PFF-triggered endocytosis is in fact leading to PFFs internalization we have repeated the experiments using a fixable version of FM1-43 (FM1-43fx) and treating the cells with Alexa-633-tagged αSyn PFFs. After 15 min of stimulation cells were fixed and imaged under confocal microscopy. Figure 3f and g shows that ~ 80% of FM1-43-positive vesicles co-localizes with Alexa-633-positive dots strongly suggesting that vesicle budding triggered by αSyn PFFs leads to internalization of αSyn PFFs in the enteroendocrine and neuronal cell lines used.

### αSyn PFFs are transferred between enteroendocrine and neuronal cells upon cell-to-cell contact

**Figure 4:**
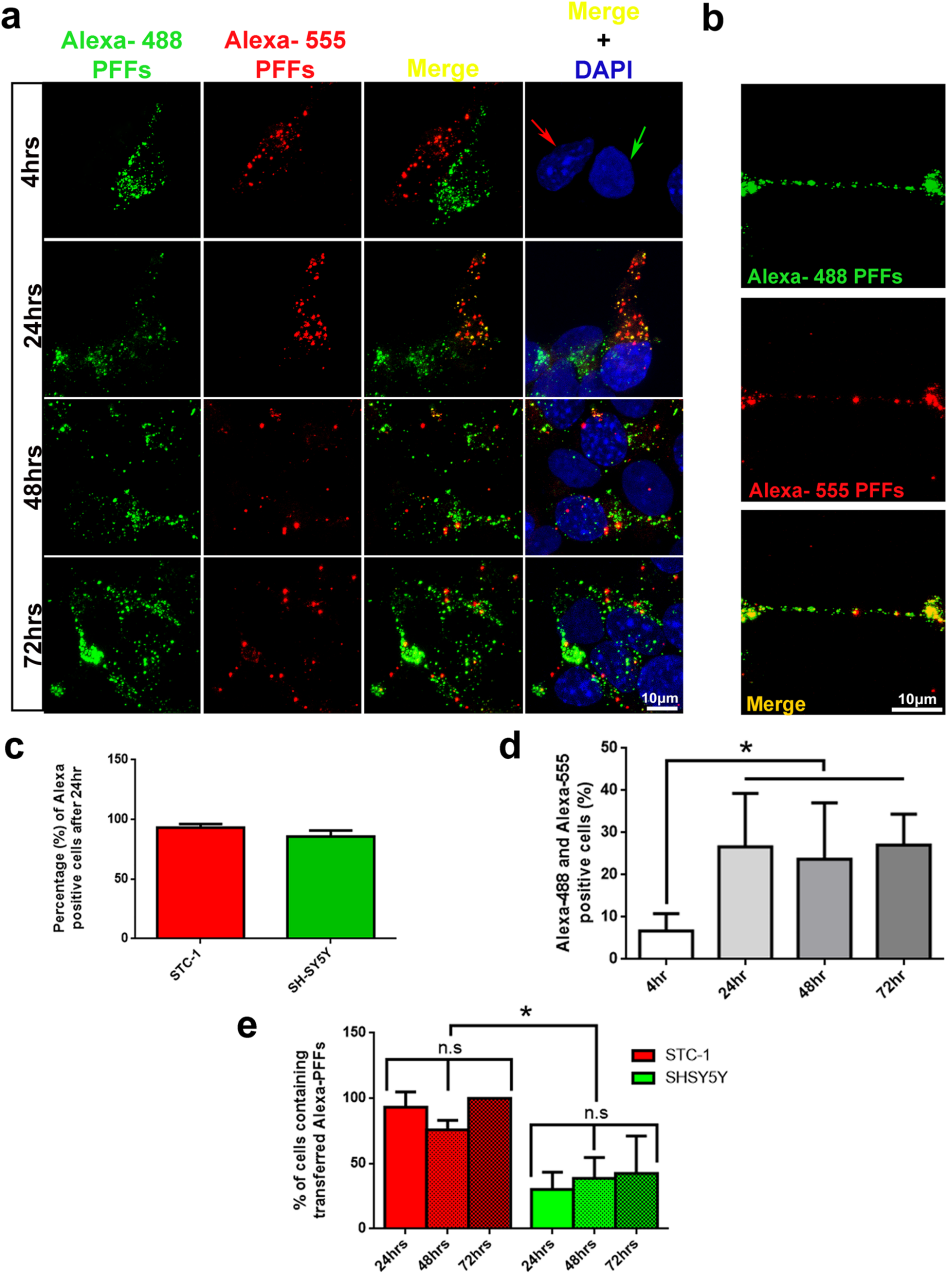
αSyn PFFs are efficiently transferred between enteroendocrine and neuronal cell lines. (**a**) Representative images of STC-1 cells loaded with Alexa-488 PFFs and SH-SY5Y cells loaded with Alexa-555 PFFs co-cultured for 4, 24, 48 and 72 h as explained in Supplementary Fig. 5. Cell types were identified by their nuclei morphology (red arrow; STC-1 nucleus. Green arrow, SH-SY5Y nucleus). Scale bar represents 10 µm (n = 3 independent experiments). (**b**) High-magnification image of a STC-1 prolonging after 72 h of co-culture showing the presence of Alexa-488 and 555-tagged PFFs. Scale bar represents 10 µm. (**c**) Graph quantification of confocal images shows the percentage of Alexa-488 and 555-positive cells after 24 h of co-culture (n = 3 independent experiments). (**d**) Graph shows percentage of Alexa-488 and Alexa-555-positive cells evaluated by flow cytometry after 4, 24, 48 and 72 h (n = 3 independent experiments; mean ± S.E.M.). (**e**) Graph quantification of confocal images shows the percentage of Alexa-488 and 555-positive cells after 24, 48 and 72 h of co-culture. STC-1 and SH-SY5Y cells were identified but their nuclear morphology and manually quantified (n = 3 independent experiments). Values are expressed as mean ± S.E.M . *p < 0.05 by two-tailed Student’s *t*-test; n.s, not significant.

EECs face the gut lumen and connect directly with αSyn-containing enteric nerve terminals in a synaptic manner forming a circuit between the gut and the ENS^15^. Although transfer of αSyn fibrils between neuronal cells have already been characterized ^24^ it is still to be demonstrated whether αSyn aggregates could be transferred from EEC to neurons. Therefore, we next assessed αSyn fibril transfer in co-cultures of STC-1 and SH-SY5Y cell lines. STC-1 cells were exposed for 24 h to Alexa-488-tagged PFFs (1 µM) and SH-SY5Y to Alexa-555-tagged PFFs (1 µM). After, cells were trypsinized to remove any membrane-bound extracellular αSyn fibrils, counted and co-cultured (1:1) for 4, 24, 48 and 72 h (Fig. 4a,b; Supplementary Fig. 5a). After co-culture period, Alexa-488, Alexa-555 and Alexa-488/555-double-positive cells were quantified by flow cytometry (Fig. 4d) and confocal microscopy (Fig. 4e). Note that because nuclear morphology is very distinct between cell types (STC-1 cells presenting nucleus with very prominent, strong DAPI-stained nucleolus in comparison to SH-SY5Y cells), it was possible to easily discriminate each cell population (Fig. 4a, arrows). As can be seen in Fig. 4c, 100% of cells co-cultured contained Alexa-tagged fibrillar αSyn (Fig. 4c). After 4 h of co-culture no transfer of αSyn PFFs between cell lines were observed (Fig. 4a). However, after 24 h, confocal microscopy and flow cytometry analysis showed that approximately 30% of STC-1 and SH-SY5Y cells started to present Alexa-488 and 555-tagged PFFs and no further transfer efficiency was observed with 48 or 72 h of co-culture (Fig. 4a,b and d). Interestingly, when we analyzed the number of STC-1 cells containing transferred PFFs (Alexa-488-positive puncta) and the number of SH-SY5Y cells containing transferred PFFs (Alexa-555-positive puncta) by confocal microscopy, we detected that while ~ 90% of STC-1 cells were positive for Alexa-488-positive puncta, only ~ 30% of SH-SY5Y cells were positive for the transferred fibrils (Alexa-555-positive puncta) (Fig. 4e). This suggests that PFF transfer is more efficient from SH-SY5Y towards STC-1 cells.

**Figure 5:**
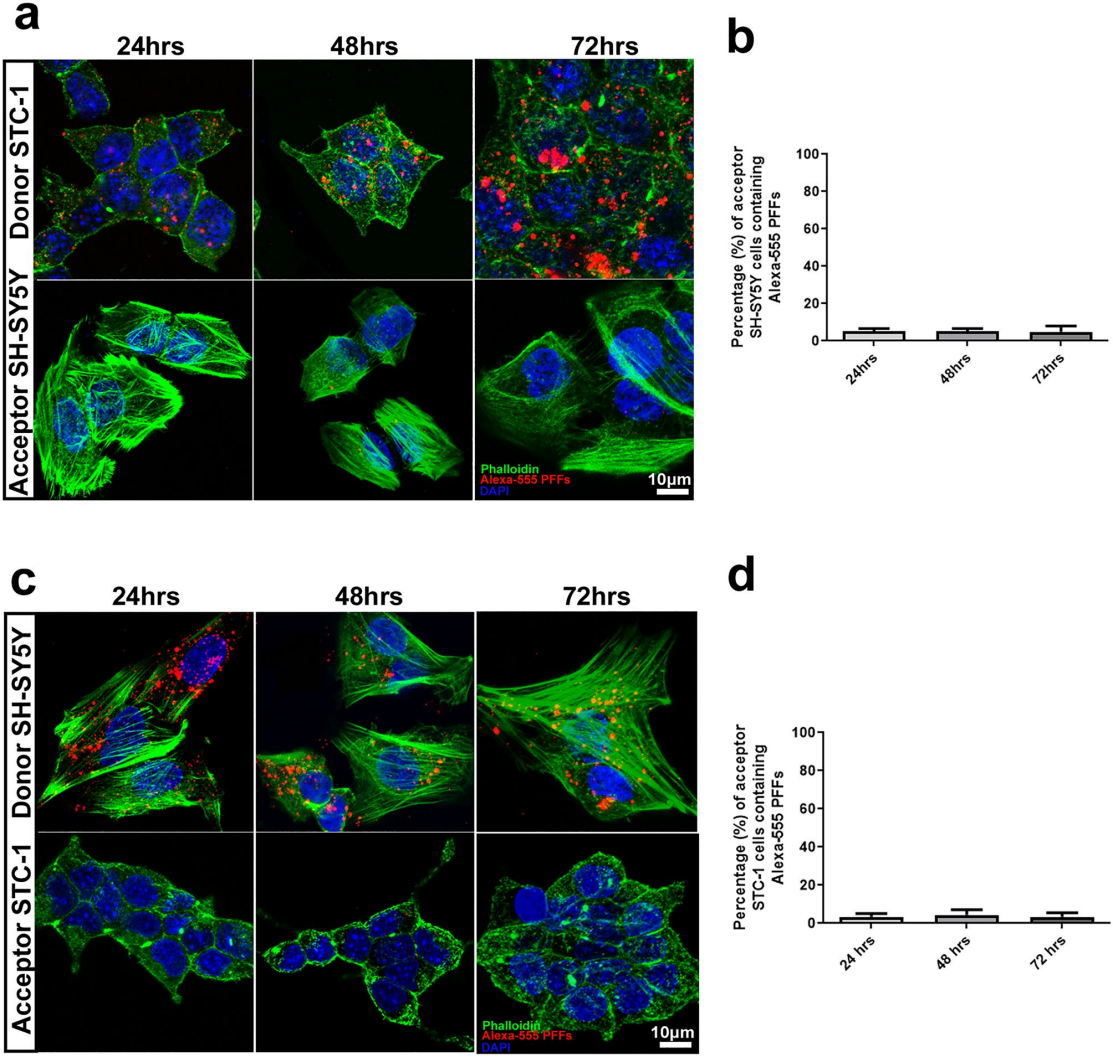
Transferring of αSyn PFFs between STC-1 and SH-SH5Y cell lines is dependent on cell-to-cell physical contact. (**a**) Representative images of donor (upper panel) STC-1 cells and acceptor SH-SY5Y cells (bottom panel) co-cultured physically separated using a filter for 24, 48 and 72 h. Donor STC-1 cells were previously loaded with Alexa-555 PFFs (red). Actin cytoskeleton is shown in green and nuclei in blue. (**b**) Quantification of the percentage of acceptor cells containing αSyn PFFs from images such as those presented in (**a)**. (**c**) Representative images of donor (upper panel) SH-SY5Y cells and acceptor STC-1 cells (bottom panel) co-cultured physically separated using a filter for 24, 48 and 72 h. Donor STC-1 cells were previously loaded with Alexa-555 PFFs (red). Actin cytoskeleton is shown in green and nuclei in blue. (**d**) Quantification of the percentage of acceptor cells containing αSyn PFFs from images such as those presented in **c**. At least 100 cells from 3 independent experiments were scored. Values are expressed as mean ± S.E.M . No significant differences were observed when evaluated by One-Way ANOVA.

To further characterize the mechanism of transfer, we performed co-cultures where the donor (STC-1 or SH-SY5Y cells) and acceptor cells (STC-1 or SH-SY5Y cells) were separated by 0.4-µm membrane pore size semipermeable to prevent contact between donor and acceptor cells, but to allow the passage of soluble factors between the two cell lines ^34,35^ (Supplementary Fig. 5b). After culturing the cells for 24, 48 or 72 h, we observed that the percentage of acceptor cells containing αSyn puncta and the number of puncta in acceptor cells were drastically decreased compared to cells co-cultured without the filtering membranes (Fig. 5a–d).

We previously showed that pathological forms of αSyn increase the intracellular levels of phosphorylated forms of αSyn in STC-1 cells (Fig. 2e). Next, we aimed to check whether these PFFs transferred from STC-1 to SH-SY5Y cells could also lead to αSyn phosphorylation in these acceptor cells. We incubated STC-1 cells for 24 h with Alexa-tagged PFFs and used Alexa-tagged αSyn monomers as a control. After checking internalization by the presence of Alexa-555 positive puncta, cells were trypsinized to remove any membrane-bound extracellular αSyn, co-cultured (1:1) for 72 h with SH-SY5Y cells and submitted to immunofluorescence for pser129-αSyn. When analyzing the fluorescence intensity levels of pser129 αSyn in acceptor cells (SH-SY5Y cells), differentiated by their nuclear morphology as previously mentioned, confocal microscopy images showed stronger deposits of pSer129-αSyn only in cells co-cultured with STC-1 cells previously treated with αSyn PFFs but not with αSyn monomers (Supplementary Fig. 6). It is worth mentioning that due to different expression of αSyn in both cell lines what leads to different pattern of antibody staining, laser intensity for imaging capture had to be set to favor observation of SH-SY5Y cells, the acceptor cells of interest in this set of experiments.

Altogether, these data indicate that in our experimental condition intercellular transfer of αSyn fibrils between enteroendocrine to neurons is favored by cell-to-cell contact and induces αSyn pathology in acceptor cells.

### Rab35 silencing impairs αSyn PFFs transfer from enteroendocrine to neuronal cells.

Next, we turned our attention to the molecular mechanisms involved in the transferring of αSyn fibrils from STC-1 cells to SH-SY5Y cells. We particularly focused on Rab proteins, small GTPases that regulate vesicle trafficking and sorting^36^. It was previously shown that Rab35 promotes tunneling nanotubes (TNT) formation and TNT-mediated vesicle transfer in CAD neuronal cell line^37^. In addition, further investigation in HeLa cells suggested that Rab35 controls a fast endocytic recycling pathway from endosomes to the plasma membrane, a mechanism that may be responsible for the sorting, transfer and spread of intracellular aggregates from one cell to another^38,39^.

**Figure 6:**
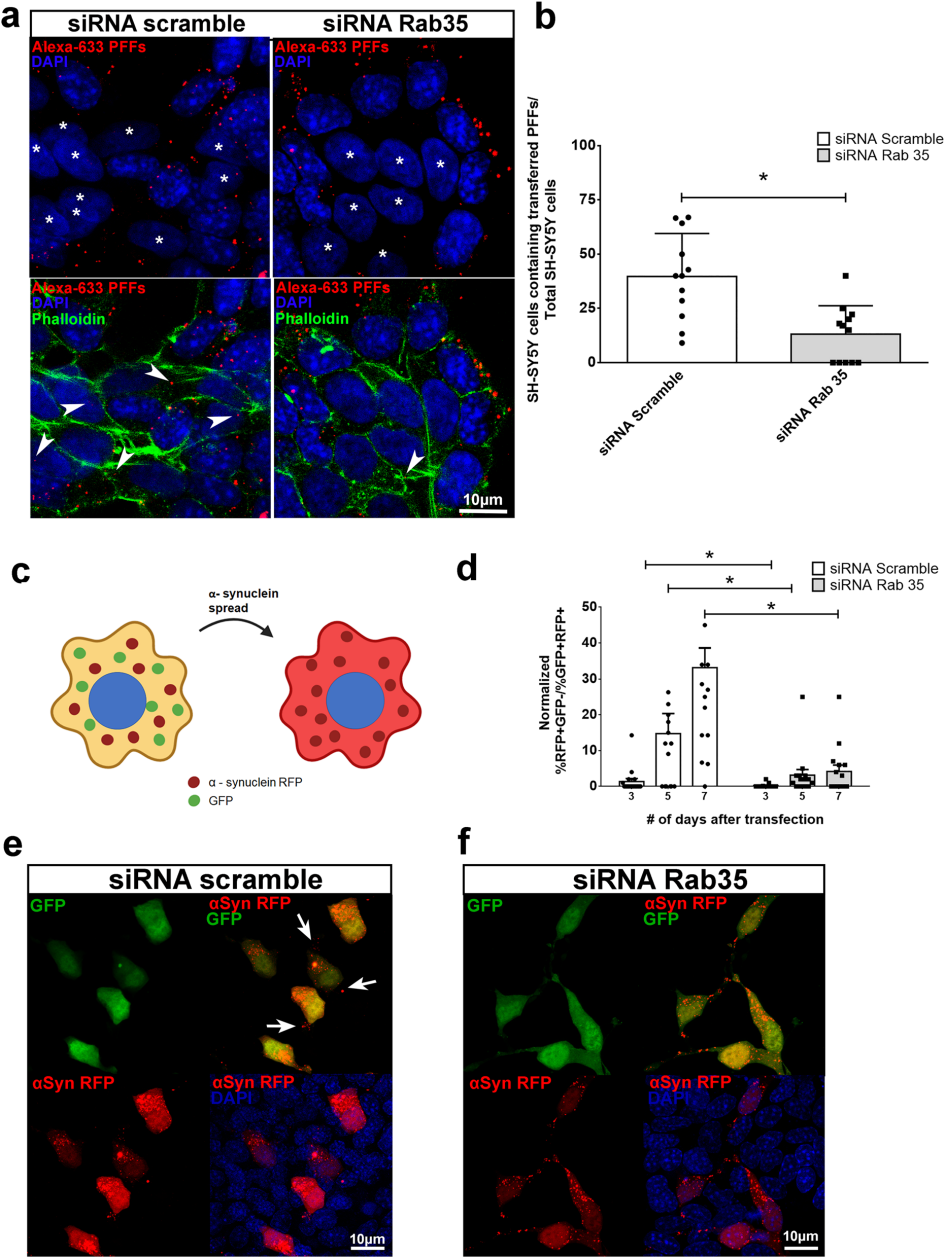
Silencing of Rab35 GTPase in STC-1 cells inhibits αSyn fibrils translocation and spreading. (**a**) Confocal images of scramble and Rab35-specific siRNA- silenced STC-1 cells co-cultured with SH-SH5Y cells (asterisks) for 24 h. STC-1 cells were silenced for 24 h, loaded with Alexa-633-tagged PFFS for the next 24 h, trypsinized and co-culture with the same number of SH-SY5Y cells for 24 h. Actin cytoskeleton was stained with phalloidin-Alexa 488 and nuclei with DAPI. Only one Z-slice of 0.25 µM is shown. (**b**) Bars show the number of SH-SY5Y cells containing transferred Alexa-633-tagged PFFs normalized by total number of SH-SY5Y cells nuclei per image. Data are expressed as mean ± S.E.M. of 12 images obtained from 4 independent experiments per condition. *p < 0.05 by two-tailed Student’s *t*-test. (**c**) Cartoon depicting the rationale behind the αSyn transfer assay used, based on the GFP-2A-αSyn-RFP construct. (**d**) Time-course experiment in which STC-1 cells were silenced with scramble or Rab35-specific siRNA for 24 h, transfected with the GFP-2A-αSyn-RFP construct, followed by collection on days 3, 5, and 7 and analysis through flow cytometry. This experiment was repeated independently 5 times. Data were normalized to the samples collected 3 days after transfection. One-way ANOVA with a test for trend, *p < 0.05. (**e**) Representative confocal images showing control STC-1 cells that were transfected with the GFP-2A-αSyn-RFP construct and is double positive for GFP and RFP, and cells that have received αSyn-RFP through transfer and are positive for RFP but negative for GFP (arrows). Scale bars, 10 μm. (**f**) Representative confocal images showing Rab35-silenced STC-1 cells that were transfected with the GFP-2A-αSyn-RFP construct and is double positive for GFP and RFP. No transferring of αSyn-RFP could be detected. For experiments portrayed in **(e)** and **(f)**, cells were cultured for 3, 5 and 7 days. Scale bars, 10 μm.

To address whether Rab35 is associated with cell-to-cell transfer of αSyn fibrils from STC-1 to SH-SY5Y cells, we silenced STC-1 cells with Rab35-specific siRNA, loaded cells with Alexa-633-tagged αSyn PFFs and co-cultured them with SH-SY5Y cells for 24 h. The effectiveness of Rab35 silencing was confirmed by immunofluorescence and western blotting (Supplementary Fig. 7). Figure 6a shows representative images of the co-culture of silenced STC-1 cells and SH-SY5Y cells. As mentioned previously, it is possible to identify each cell line by nuclear morphology (nuclei of SH-SY5Y cells marked with asterisks). When we quantified the number of SH-SY5Y cells containing Alexa-633-positive PFFs relative to the total number of SH-SY5Y on each image we detected a reduced number in the group co-cultured with Rab35-silenced STC-1 cells suggesting that this GTPase play a role in the transfer of αSyn fibrils between cells (Fig. 6a,b).

To confirm that the effects observed was not due to a deficit in PFFs internalization due to Rab35 silencing but specifically due to impairment in PFFs transfer from silenced STC-1 cells to SH-SY5Y cells, we used a GFP-2A-αSynuclein-RFP reporter ^22,23^. In this construct, the translated protein is cleaved at the 2A peptide into two proteins: GFP and αSyn-RFP. αSyn-RFP is then transferred to neighboring cells without concomitant transfer of GFP (Fig. 6c). Thereby, donor and recipient cells can be identified by their colors: the cells expressing αSyn through transfection are positive for GFP and RFP while the cells that receive αSyn through transfer are positive for RFP only. In this case, the STC-1 cells were co-transfected with Rab35-specific siRNA and the plasmid DNA construct. After 48 h of expression, we monitored the number of STC-1 cells positive for RFP (RFP^+^) but negative for GFP (GFP^-^). In a time-course experiment with control cells, we found an increase over time of the number of RFP^+^GFP^−^ cells that had received αSyn-RFP through transfer. However, the rate of αSyn-RFP transfer is greatly reduced in Rab35-silenced cells (Fig. 6d–f).

Therefore, with both approaches used, we confirmed that silencing of Rab35 in the STC-1 enteroendocrine cell line diminishes the transfer rate of αSyn fibrils between cells.

### Rab35 silencing redirects αSyn fibrils to lysosomal compartments.

Rab35 plays important roles in endosomal recycling and actin filament remodeling^40,41^. In addition, it is proposed that Rab35 phosphorylation causes dyshomeostasis of αSyn aggregate trafficking through the endo-lysosomal pathway^42^. Considering its role in endosomal recycling, it is tempting to speculate whether silencing of Rab35 would direct the internalized αSyn aggregates to the lysosomal degradation pathway.

**Figure 7:**
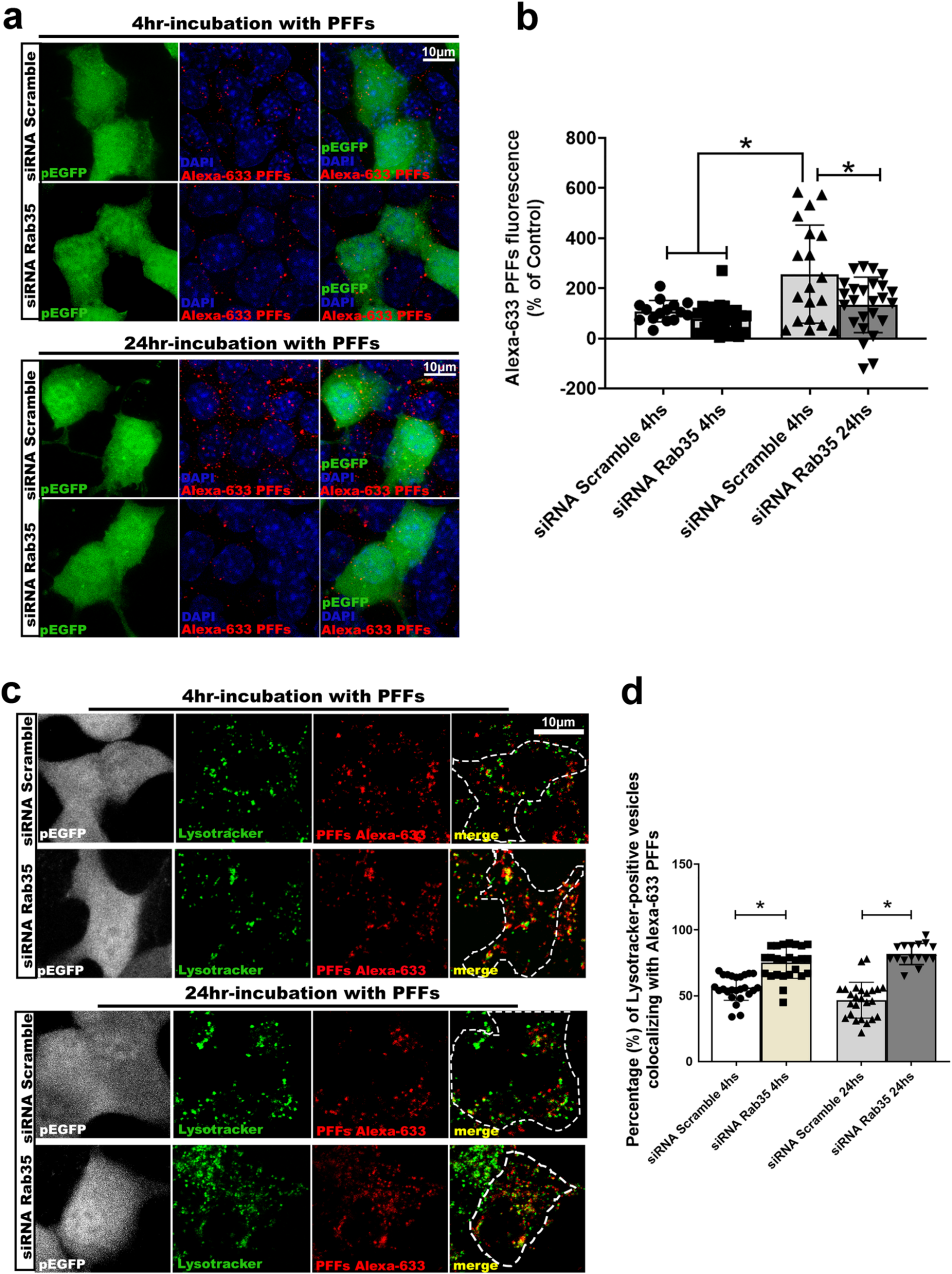
Silencing of Rab35 GTPase in STC-1 cell redirects αSyn PFFs to lysosomes. **(a**) Representative images of STC-1 cells co-transfected with pEGFP and a control scramble/or a Rab35-specific siRNA and incubated for 4 (upper panel) or 24 h (bottom panel) with αSyn PFFs-Alexa 633-tagged. (**b**) Graph shows the quantification of Alexa-633 fluorescence in pEGFP-positive cells like the ones represented in **(a)**. It can be observed that after 24 h of incubation with PFFs, Rab35-silenced STC1 cells displays a reduced level of Alexa-633 fluorescence but no difference was noticed at 4 h-incubation period. Values are expressed as mean ± S.E.M. One-way ANOVA with a test for trend, *p < 0.05. This experiment was repeated independently 5 times and at least 20 images were analyzed. (**c**) Representative images of STC-1 cells co-transfected with pEGFP and a control scramble/or a Rab35-specific siRNA and incubated for 4 (upper panel) or 24 h (bottom panel) with Alexa-633-tagged αSyn PFFs-. After incubation period, cells were stained with Lysotracker. (**d**) Graph shows the quantification (%) Lysotracker-positive vesicles (green) co-localizing with Alexa-633 PFFs (red) in pEGFP-positive cells (gray) like the ones represented in **(c)**. Values are expressed as mean ± S.E.M. One-way ANOVA, *p < 0.05. This experiment was repeated independently 5 times and at least 20 images were analyzed. Scale bars, 10 μm.

When we silenced Rab35 and incubated STC-1 cells with αSyn PFFs for 4 and 24 h, we observed that the internalized level of PFFs was significantly reduced when compared to control cells. Interestingly, no differences were observed immediately after 4 h of incubation (Fig. 7a,b). Since silencing of Rab35 also diminishes the transfer of PFFs between cells, this observation reinforced our hypothesis that somehow silencing of Rab35 could be leading αSyn PFFs to lysosomal degradation.

Incubation of control STC-1 cells with PFFs for 1, 4 and 24 h showed that, after 4 h, ~ 60% of αSyn puncta co-localized with Lysotracker positive vesicles (Supplementary Fig. 8). Extending this observation to Rab35-silenced STC-1 cells, we performed a co-transfection with Rab35-specific siRNA and a plasmid expressing GFP to visualize silenced cells. Incubation of the cells with PFFs for 4 and 24 h showed that the co-localization rate of Alexa-633-positive puncta with Lysotracker-positive vesicles was considerably higher in Rab35-silenced cells when compared to control immediately after 4-h-incubation period. Taken together, these results support the notion that Rab35 plays an important role in the transfer mechanism of αSyn fibrils initiated in enteroendocrine cells and that inhibition of Rab35 increases the clearance of αSyn fibrils by redirecting them to the lysosomal compartment.

## Discussion

Although genetic risks have been identified, approximately 90% of diagnoses of PD are idiopathic^43^, yet the factors that cause pathogenesis remain unclear. Interactions between genetic and environmental factors, such as toxins, likely trigger pathogenesis by stimulating αSyn oligomerization and aggregation. Interestingly, αSyn pathology in PD is not limited to the brain. This notion is supported by observations of αSyn-containing inclusions in peripheral tissue, including the ENS, from patients with PD and from otherwise healthy individuals. In addition, the idea of pathological propagation of αSyn has gained much attention since the host-to-graft propagation of αSyn-positive Lewy-like pathology in long-term mesencephalic transplants in PD was shown^44,45^. The transfer of αSyn between co-cultured neuronal cells in vitro^24,46^ and the indication of bidirectional αSyn propagation via the vagus nerve have also been demonstrated^47^. However, since the rise of the EECs as a possible site for the outcome of αSyn pathology, it is still to be demonstrated whether/how αSyn aggregates could be transferred from the EECs to neuronal cells.

The EECs are directly influenced by the gut lumen microenvironment and in contact with neurons from the ENS. Therefore, it is reasonable to hypothesize that αSyn misfolding might start in EECs^16^, propagate from the gut epithelium to the ENS and then be transferred to the CNS. Characterizing this process is critical for understanding the pathology and for the design of strategies aimed at interfering with the propagation component of PD.

Although EECs are located in the gut epithelium, they behave differently from regular enterocytes being able to reside in the mucosa for months instead of being replaced every 3–5 days^48^. Therefore, EEC-neuronal connection is likely to be long lived enough for conveying pathological events.

When thoroughly characterizing the internalization of PFFs by EECs, we showed that αSyn PFFs initiate intracellular Ca^2+^ signaling and activate endocytic events in the enteroendocrine cell line STC-1 sharing these commonalities with neuronal cell line SH-SY5Y. The direct observation of endocytic vesicle budding in real time we offer here strengthens previous findings reporting that the presence of dynamin inhibitors (that block membrane scission) blocks fibrils internalization, suggesting the involvement of the endocytic pathway in this process^49,50^. In addition, we showed that the spread of αSyn PFFs from enteroendocrine to neuronal cells is dependent on physical cell-to-cell contact and Rab35 GTPase.

It was previously reported that impaired cell-to-cell contact of neuronal cells in vitro reduced the transfer of αSyn fibrils^24^ . In addition, although secretion of αSyn fibrils was once suggested as a major path for αSyn transfer, physical contacts between cells significantly increased transfer^46,51,52^. This indicates that either physical cell-to-cell contact or close proximity between the cells is essential for efficient transfer of αSyn fibrils. It is worth mentioning that the αSyn transfer mechanisms between EECs and neuronal cells has never been shown. Using αSyn PFFs with different fluorophores, we have also demonstrated here that intercellular transfer of αSyn fibrils can happen in a retrograde fashion from the nervous system onto EECs. This notion is supported by a recent study performed in vivo in which the evidence of αsyn propagation to the heart via the vagus nerve came to light^47^. Interestingly, in our experimental condition, the spread of αSyn fibrils from neuronal cells to EECs was shown to be even more efficient than the opposite route. Therefore, whether the presence of αSyn aggregates in the EECs is a secondary event from αSyn pathology started in the ENS or CNS could have significant importance for our understanding of role of EECs in PD.

Our work also reinforces the role of Rab35 as a key molecular character in αSyn propagation. Rab35 has been recognized as the regulator of several important events of neuronal physiology including neurite outgrowth^53^, cytokinesis^54^, cell polarity^41^, and exosome secretion^55^. Findings from a previous study mirror our own, as that study reported that propagation of αSyn between neurons is augmented due to Rab35 activation^42^. However, according to our results, this seems to be a shared mechanism between neurons and EECs since Rab35-silenced EECs exhibited increased levels of internalized Alexa-633 PFFs after 24 h when compared to control cells. Moreover, we showed that Rab35 is mainly involved in the propagation of Rab35 and not in internalization, hijacking the internalized αSyn aggregates from the lysosomal degradation what leads to amplification of aggregates and continuous propagation (Fig. 7). Although it is known Rab35 role in endosomal recycling and actin filament remodeling^40,41^ how Rab35 activation precisely regulates αSyn propagation is still unclear.

In summary, our findings suggest that EECs represent a key element in the gut-brain hypothesis for the outcome and progression of αSyn pathology. These cells have shown to be able to internalize and propagate αSyn fibrils to neighboring neuronal cells in a manner dependent on cell-to-cell contact and Rab35 similarly to what have been previously shown to neurons. This represents a major breakthrough in understanding the mechanisms underlying the progression of synucleinopathies by shifting the focus of PD etiology to the peripheral nervous system and putting the Rab35 GTPase as a target to be considered in future pharmacological intervention to prevent PD progression.

## Data and code availability

All data are available within the article or its supplementary materials. Any additional information is available from the Corresponding author on request.

## Supplementary Information

Supplementary Information 1.

Supplementary Video 1.

Supplementary Video 2.

## Data Availability

All developed expression plasmids produced in this study can be made available upon request to the Corresponding Author.
