# Supplementary material for: Transcellular propagation of fibrillar α-synuclein from enteroendocrine to neuronal cells requires cell-to-cell contact and is Rab35-dependent
Source: Sci Rep. 2022 Mar 9;12:4168. doi: 10.1038/s41598-022-08076-5 (PMC8907230; doi:10.1038/s41598-022-08076-5)
Supplement: Supplementary file 1 — Supplementary Information 1. [file 41598_2022_8076_MOESM1_ESM.docx]

**Scientific Reports**

**Supplementary Material**

**Transcellular propagation of fibrillar α-synuclein from enteroendocrine to neuronal cells requires cell-to-cell contact and is Rab35-dependent**

Paulla Vieira Rodrigues^1,2^; João Vitor Pereira de Godoy^1,2^; Beatriz Pelegrini Bosque^1,2^; Dionísio Pedro Amorim Neto^1,2^; Katiane Tostes^1^; Soledad Palameta^1^; Sheila Garcia-Rosa^1^; Celisa Caldana Costa Tonoli^1^; Hernandes Faustino de Carvalho^2^; Matheus de Castro Fonseca^1,2*^

^1^ Brazilian Biosciences National Laboratory (LNBio), Brazilian Center for Research in Energy and Materials (CNPEM), Campinas, São Paulo, Brazil.

^2^ Department of Structural and Functional Biology, State University of Campinas, Campinas, São Paulo, Brazil

**To whom correspondence should be addressed:**

Matheus de Castro Fonseca,

10000 Giuseppe Maximo Scolfaro St., 13083-100, Campinas, São Paulo, Brazil. Email: [hp.matheus@gmail.com](mailto:hp.matheus@gmail.com); Phone: +5535121119

**Present address:** 1200 E. California Boulevard, Laboratory of Sarkis Mazmanian, Division of Biology and Biological Engineering, California Institute of Technology, CA, USA.

**Supplementary Figures**


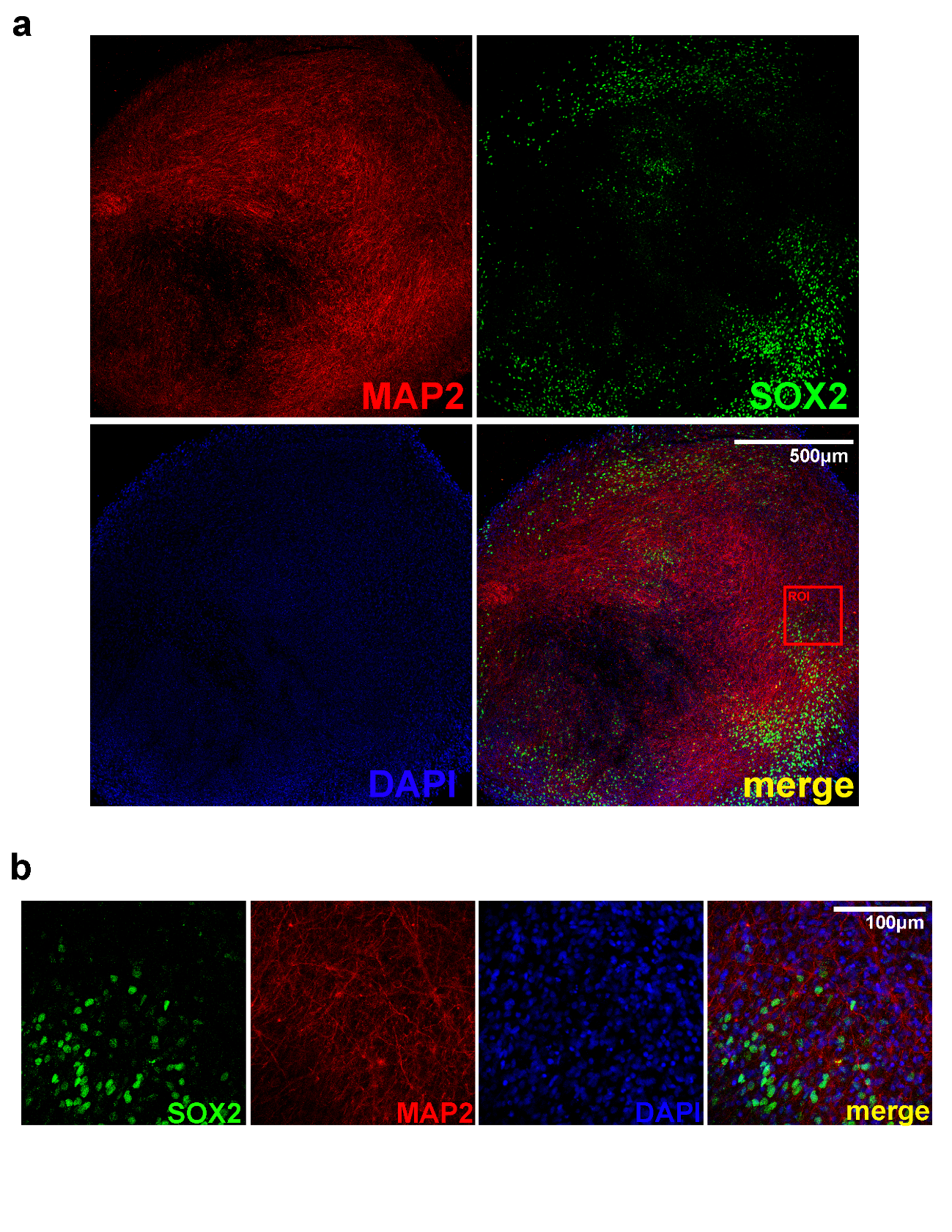


**Supplementary Figure 1. Characterization of iPSC-derived human brain organoids. (a)** Immunofluorescence of a 150µm-thick section of a brain organoid after 60 days of differentiation. MAP2 (red), SOX2 (green) and DAPI (blue) staining are shown. Scale bar, 500 μm. **(b)** High-magnification image of the ROI shown in **(a)** shows well-differentiated neuron immunolabeled for MAP2 (red) and proliferative cells immunolabeled for SOX2 (green). Nuclei are stained with DAPI (blue). Scale bar, 100 μm.


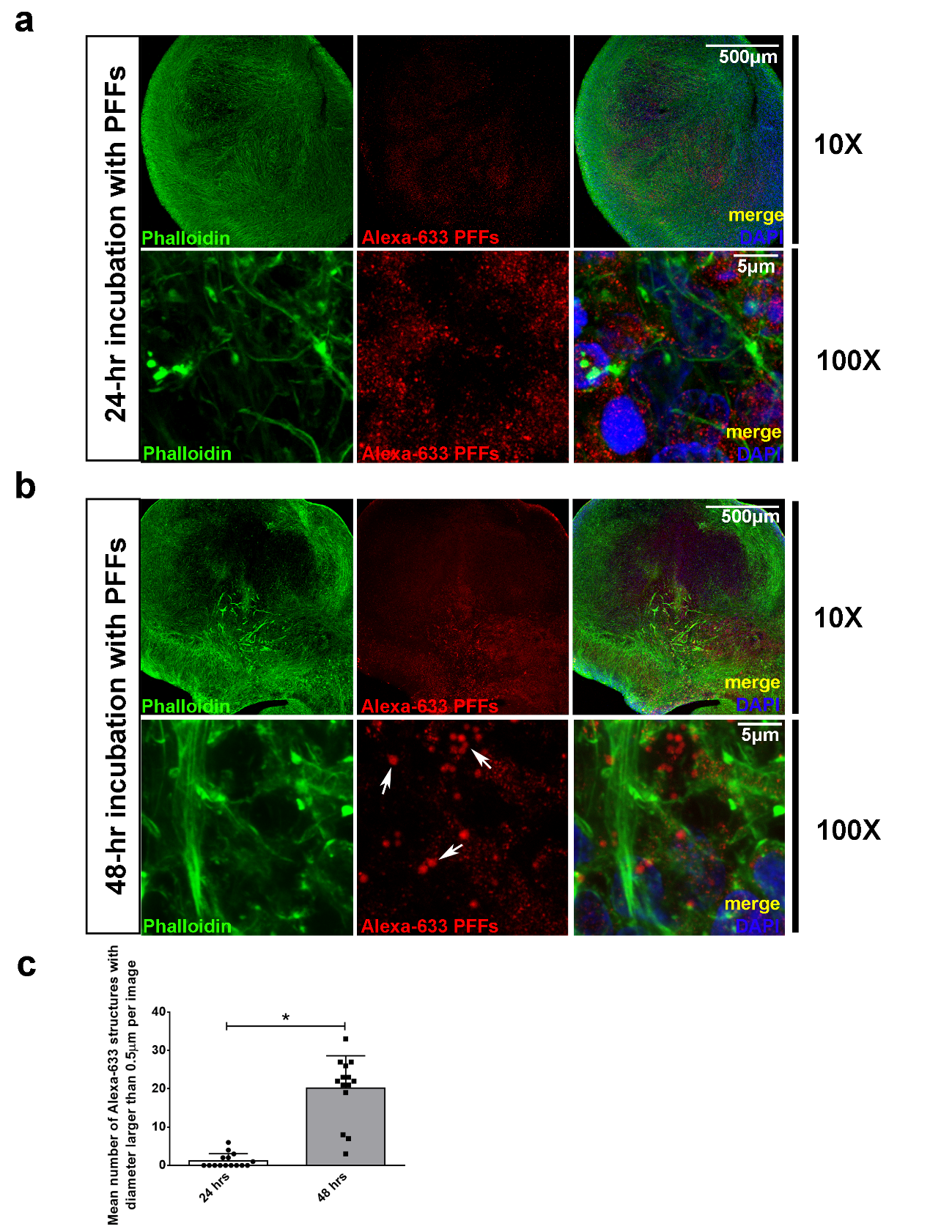


**Supplementary Figure 2. αSyn PFFs are internalized by human brain organoids and initiate a time-dependent aggregation process. (a)** Representative confocal images (10 and 100x magnification) of 150-μm-thick brain organoid after 24hr-incubation with Alexa-633-tagged αSyn PFFs (red). Actin cytoskeleton is labeled with phalloidin (green) and nuclei with DAPI (blue). Scale bars, 500 and 5 μm. **(b)** Confocal images (10 and 100x magnification) of 150-μm-thick section of a brain organoid after 48 h of incubation with Alexa-633-tagged αSyn PFFs (red). Actin cytoskeleton is labeled with phalloidin (green) and nuclei with DAPI (blue). Arrows point out Alexa-633-positive aggregates larger than 0.5μm Scale bars, 500 and 5 μm. **(c)** Graphic quantification of the mean number of Alexa-633-positive structures with diameter larger than 0.5µm per analyzed image. Values are expressed as mean ± S.E.M. *p < 0.05 by two-tailed Student’s t-test. This experiment was repeated independently with 3 organoids from different batches and at least 14 images per group (24 and 48 h) were analyzed. Scale bars, 5 and 500 μm.


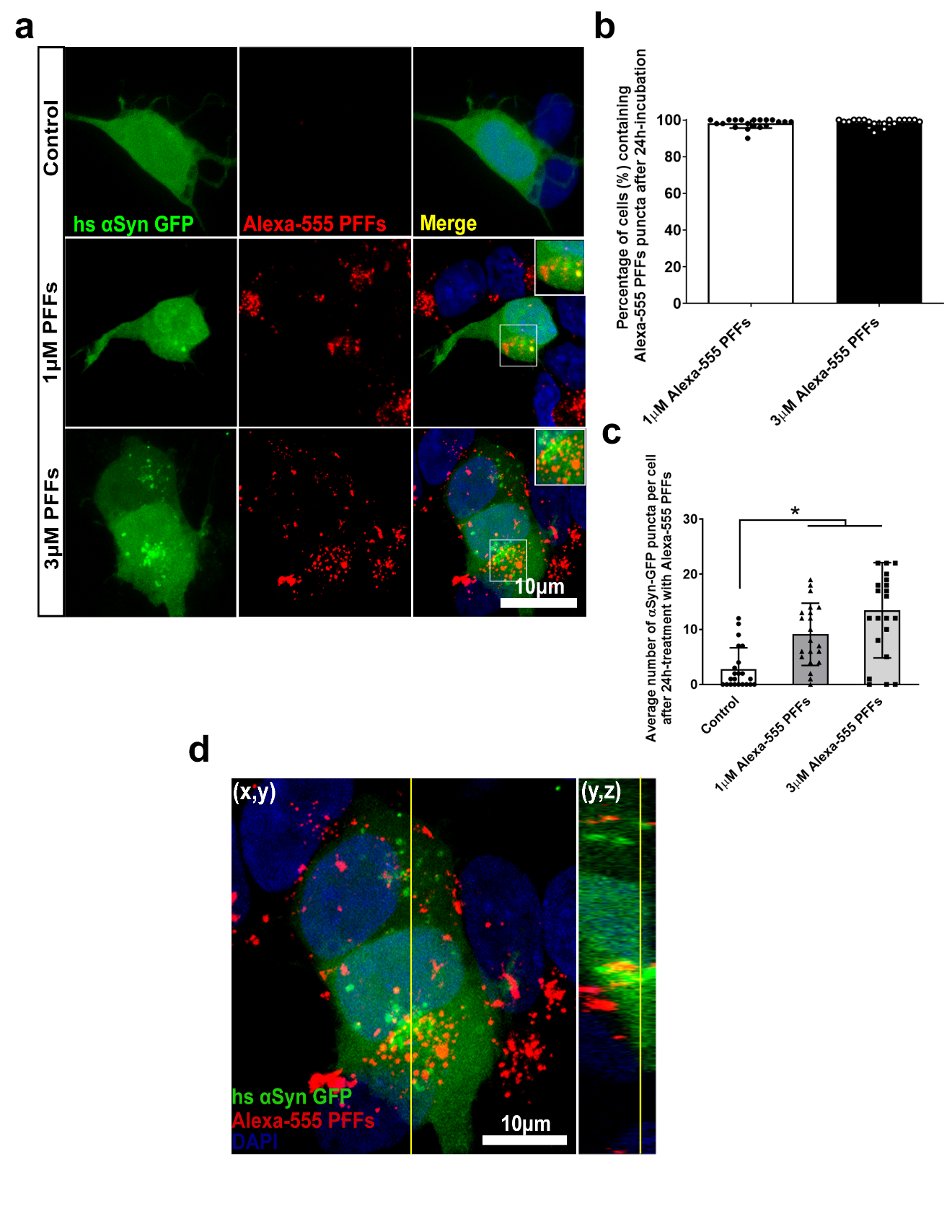


**Supplementary Figure 3. αSyn PFFs induce the aggregation of the soluble αSyn protein in SH-SY5Y cells. (a)** Representative confocal images showing GFP-αsyn-transfected cells (in green) treated with 0 (control), 1 or 3µM of fluorescent Alexa-555 αSyn fibrils (in red) for 24 h . Inserts show αSyn PFFs co-localized with GFP- αSyn puncta. Scale bars represent 10 µm. Nuclei are stained with DAPI (blue). n = 3 independent experiments. **(b)** Quantification of the number (%) of GFP-αSyn positive cells containing Alexa-555 PFFs when cells were incubated with 1 or 3 µM of PFFs from 3 independent experiments. Data are shown as mean ± S.E.M. **(c)** Graph shows the mean number of GFP-αSyn puncta in transfected cells (in green) treated with 0 (control), 1 or 3µM of fluorescent Alexa-555 αSyn fibrils (in red) for 24 h. The dots represent the mean ± S.E.M number of 3 independent experiments in which at least 25 cells per experiment were analyzed. **(d)** High magnification image of a GFP-αSyn positive SH-SY5Y cell treated with 1 µM of fluorescent Alexa-555 αSyn fibrils (in red) shows co-localization of GFP-αSyn positive puncta with Alexa-555-PFFs. Orthogonal slice (y,z) show that this event happens within the cell cytoplasm. Scale bar, 10 µm.


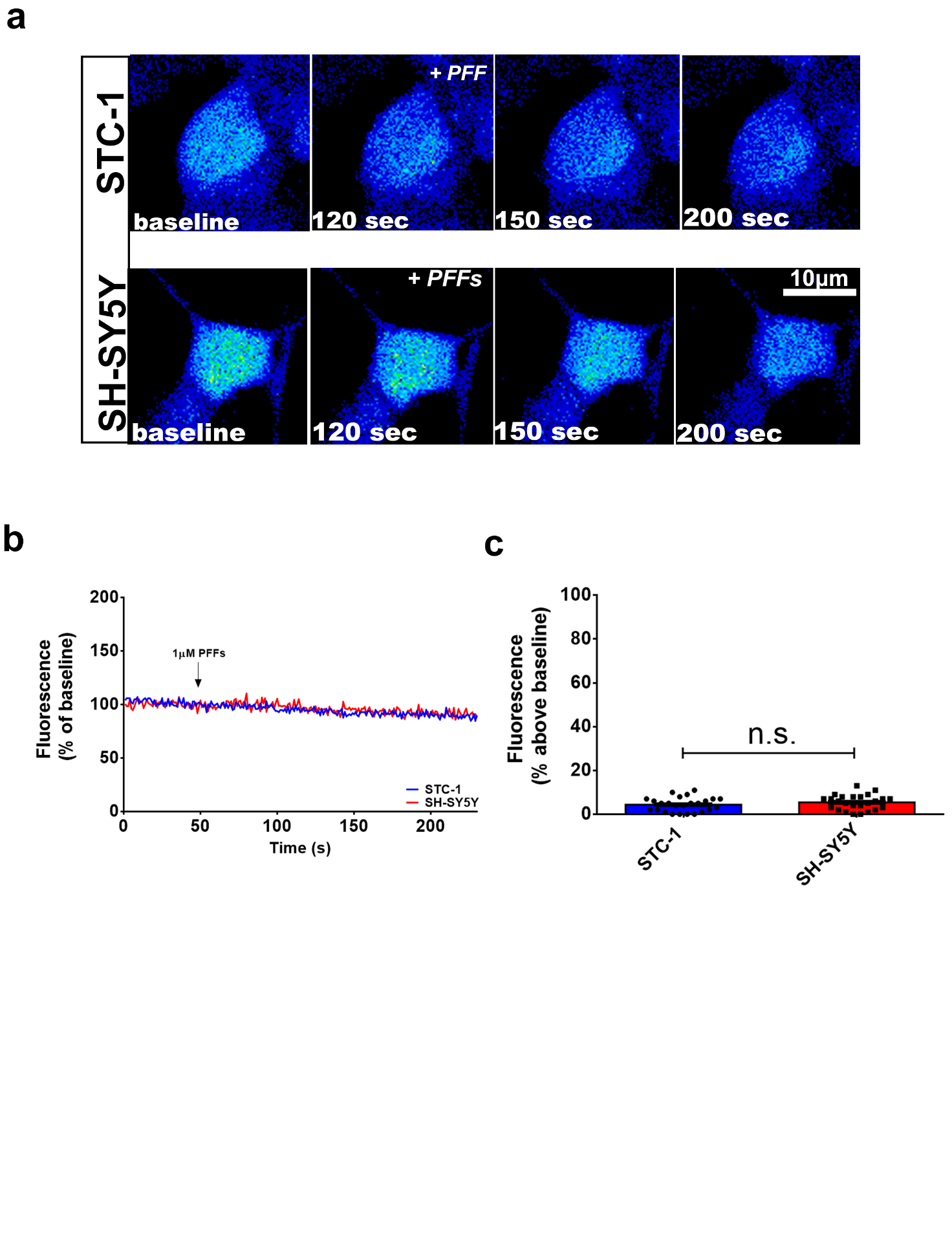


**Supplementary Figure 4: αSyn PFFs-induced Ca^2+^ response is dependent on extracellular Ca^2+^ sources. (a)** Confocal microscopy imaging of STC-1 (upper panels) and SH-SY5Y (bottom panels) cells incubated with Fluo-4/AM (6μM) and stimulated with 1 µM αSyn PFFs in Ca^2+^-free HEPES buffer solution (Scale bar: 10 μm). **(b)** Representative time‐course of total Ca^2+^ signal. Arrow indicates the moment when PFFs were applied. **(c)** Quantification of the peak fluorescence following stimulation with 1 µM αSyn PFFs in Ca^2+^-free HEPES buffer solution. (Error bars indicate the media ± SEM; n= at least 25 cells for each group, * p< 0.05 by unpaired Student’s t-test).


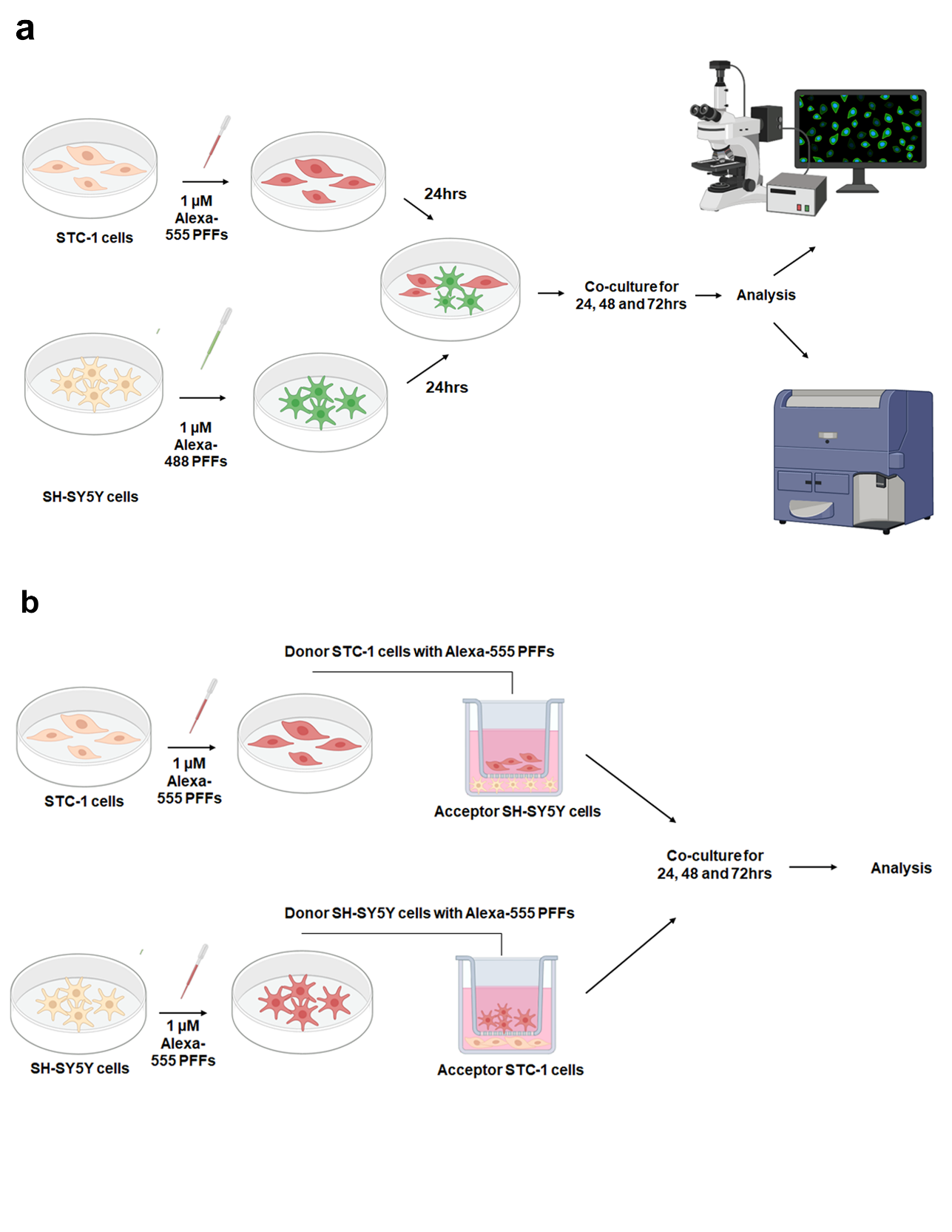


**Supplementary Figure 5. Experimental set-up used to assay transfer of αSyn PFFs through cell-to-cell contacts (a) and physical separation (b) in STC-1 and SH-SY5Y co-cultures.** In the cell-to-cell contact assay **(a)**, STC-1 cells were loaded with αSyn PFFs-Alexa-555 and the SH-SY5Y cells were loaded with αSyn PFFs-Alexa-488. After 24hrs of internalization, cells were co-cultured for 24, 48 and 72h before being analyzed by flow cytometry and immunofluorescence. In the cell-to-cell contact impairment (No physical contact) assays **(b)**, we used 0.4µm-transwell filters. The donor cells (already loaded with αSyn PFFs-Alexa 555) were plated on top of the transwell filter whilst the receptor unloaded cells were previously seeded on coverslips on the bottom of a 6-well plate. Both STC-1 and SH-SY5Y cells were used as donor or receptors. Cells were analyzed confocal microscopy after 24, 48 and 72h of co-culture. Figure was created with BioRender.com.


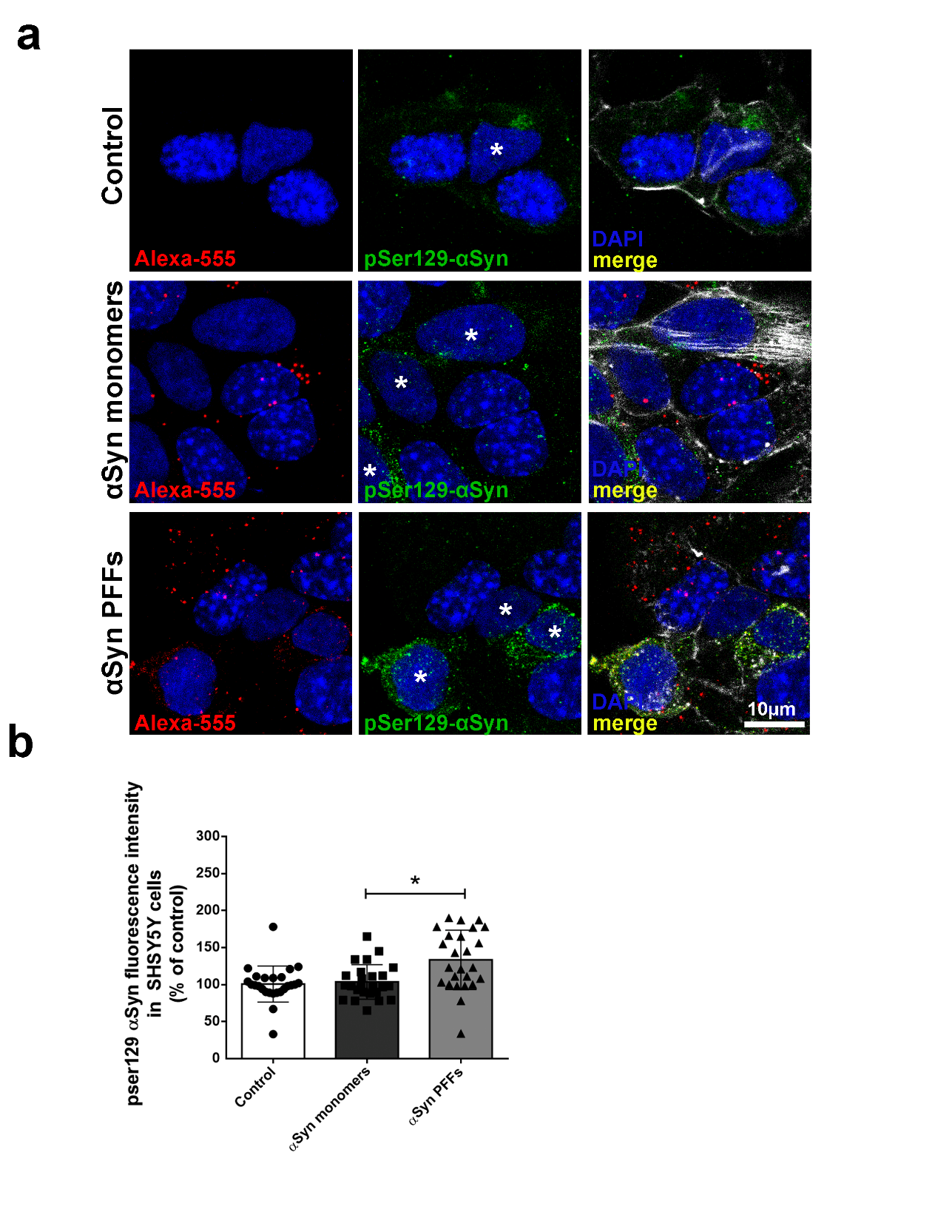


**Supplementary Figure 6. Transferred αSyn PFFs induce αSyn phosphorylation in acceptor SH-SH5Y cells. (a)** Representative images of control STC-1 cells (upper panel), STC-1 cells loaded with Alexa-555 αSyn monomers (middle panel) or Alexa-555 αSyn PFFs (bottom panel) and co-cultured for 72 h with SH-SY5Y cells (marked with asterisks), as explained in Supplementary Figure 5. pser129- αSyn immunofluorescence labeling is shown in green. Actin cytoskeleton was stained with phalloidin-Alexa-647 (gray). Scale bar represents 10 µm. **(b)** Fluorescence intensity analysis revealed increased levels of pser-129 αSyn in acceptor cells incubated with αSyn-PFFs-loaded STC-1 cells but not cells loaded with αSyn monomers. Values are expressed as mean ± S.E.M. *p < 0.05 by mean ± S.E.M. One-way ANOVA from three independent experiments in which at least 25 individual cells were analyzed.


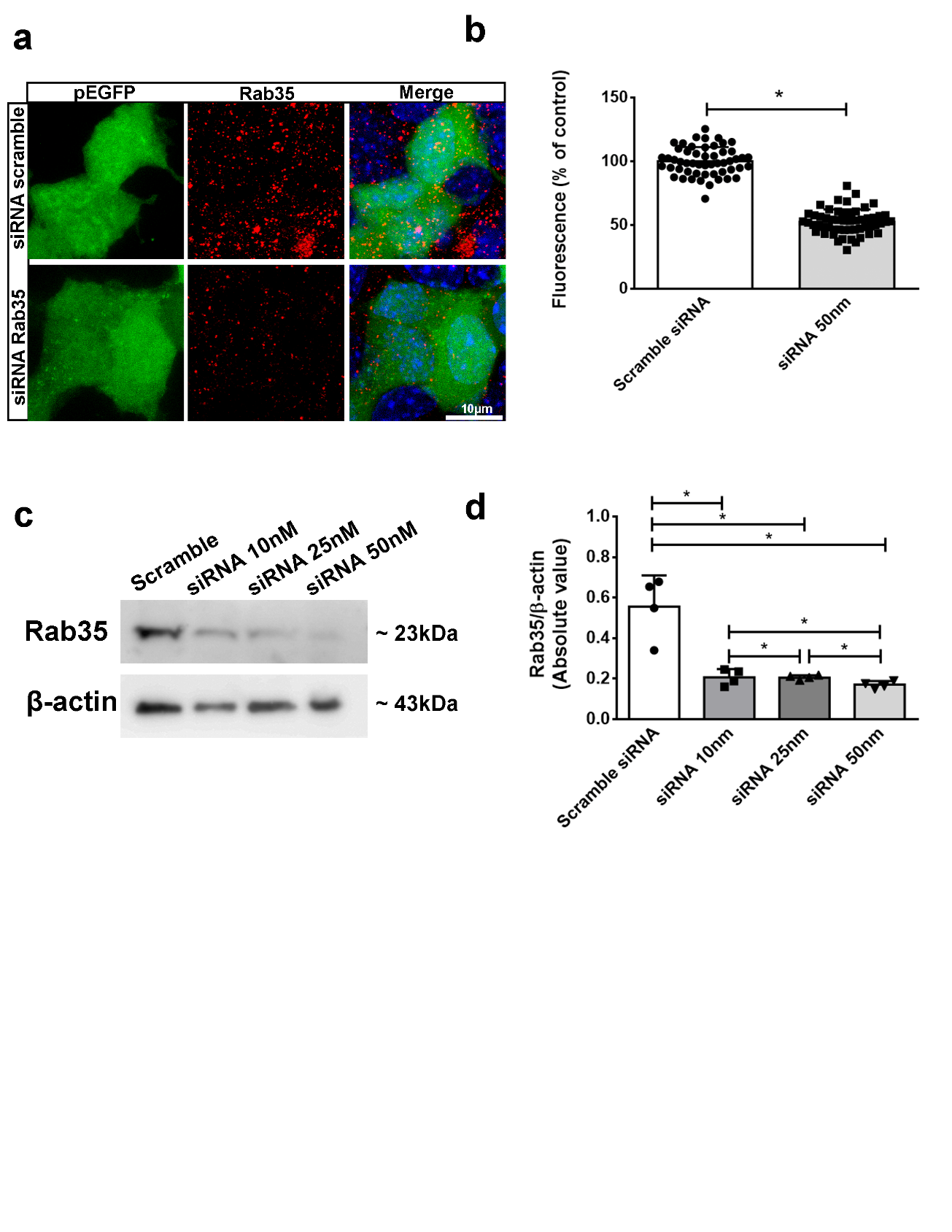


**Supplementary Figure 7. Characterization of siRNA-mediated Rab35 silencing in STC-1 cells. (a)** Confocal images of immunostained STC-1 cells co-transfected with pEGFP plasmid (green images) and 10nm of a scramble control or Rab35-specific siRNA after 24 hrs. Rab35 was immunolabeled and is shown in red. Scale bar represents 10 µm. **(b)** Graph shows the quantification of Rab35 fluorescence in pEGFP-positive cells like the ones represented in **(a)**. Values are expressed as mean ± S.E.M. *p < 0.05 by two-tailed Student’s t-test. This experiment was repeated independently at least 3 times and at least the mean fluorescence of GFP-positive cells from 20 images were analyzed. **(c)** Western immunoblotting to confirm the silencing of Rab35 with different siRNA concentrations (10, 25 and 50 nm). Densitometric analysis are shown on **(d).** Values are expressed as mean ± S.E.M. *p < 0.05 by mean ± S.E.M. One-way ANOVA. This experiment was repeated independently at least 3 times.


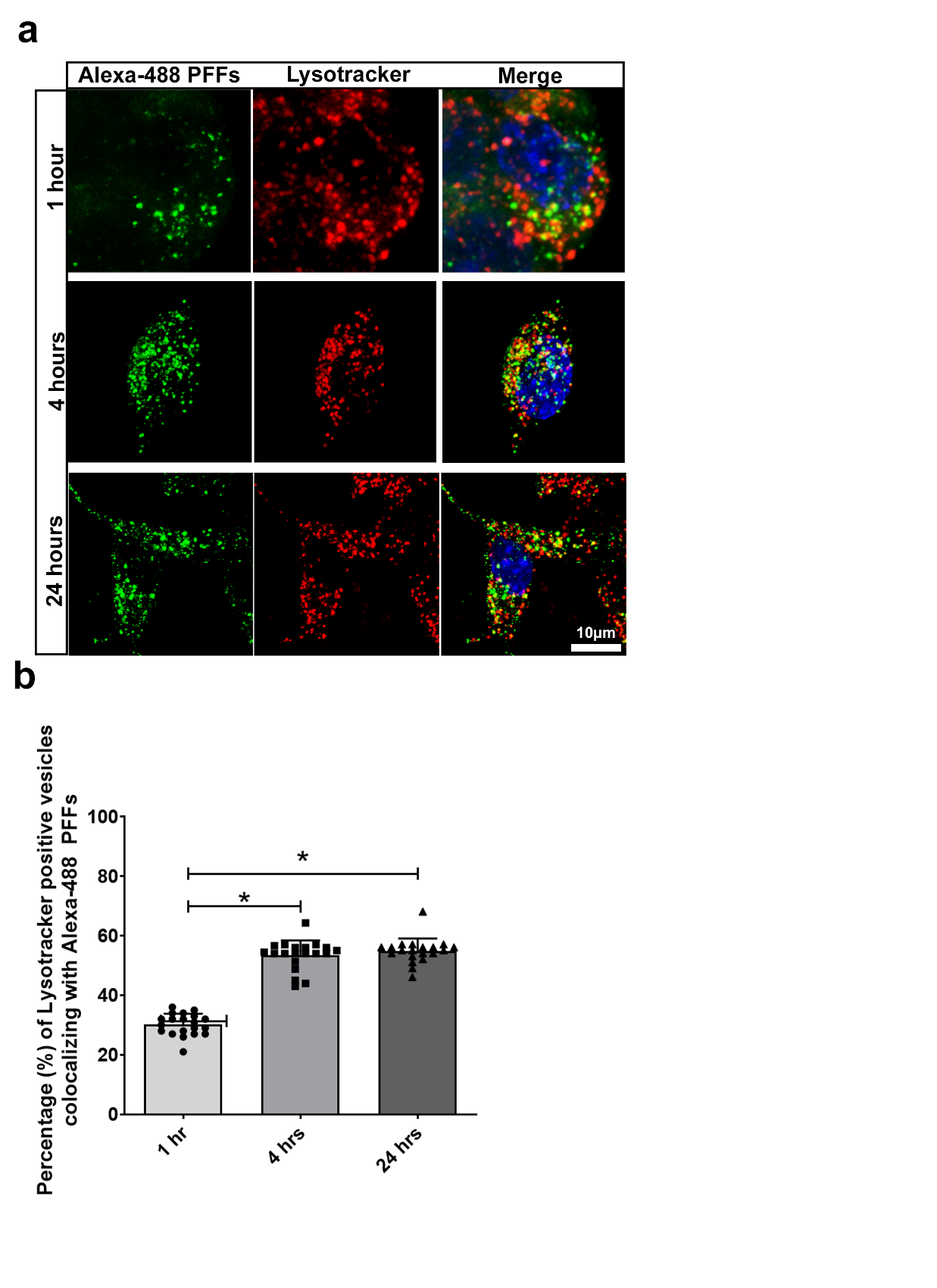


**Supplementary Figure 8. Internalized αSyn PFFs are found in lysosomal vesicles in the cytosol of STC-1 cells. (a)** Representative images of co-localization of Alexa-488-positive αSyn puncta (in green) with lysosomal vesicles (Lysotracker red DND-99) (in red) after 4, 24 and 48hrs of treatment. Scale bar represents 10 µm. **(b)** The percentage of co-localization of LysoTracker-positive vesicles with αSyn fibrils revealed that 80% of internalized PFFs co-localizes with lysosomes after 24hrs. Values are expressed as mean ± S.E.M. *p < 0.05 by mean ± S.E.M. One-way ANOVA from three independent experiments.

**
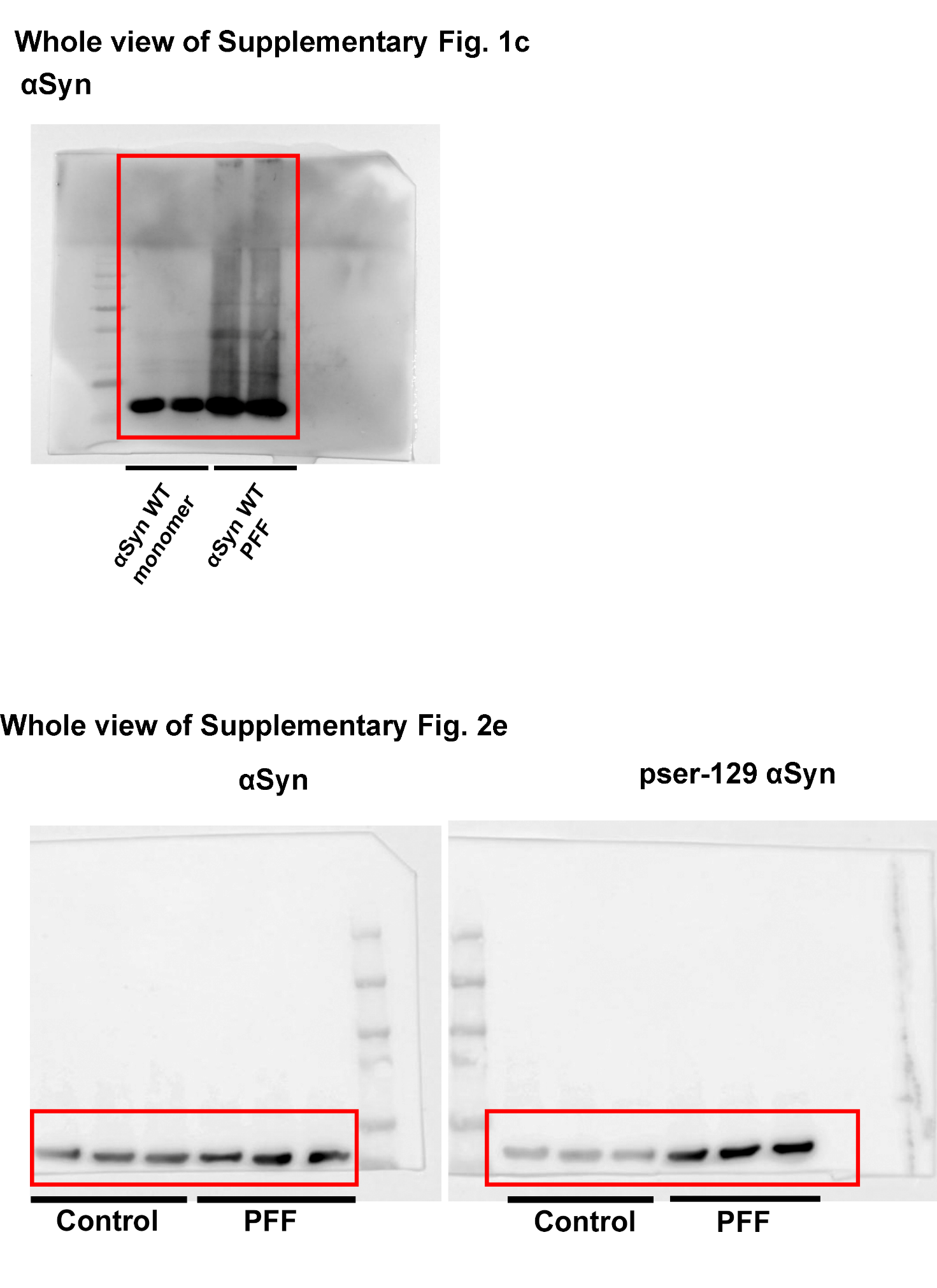
**

**
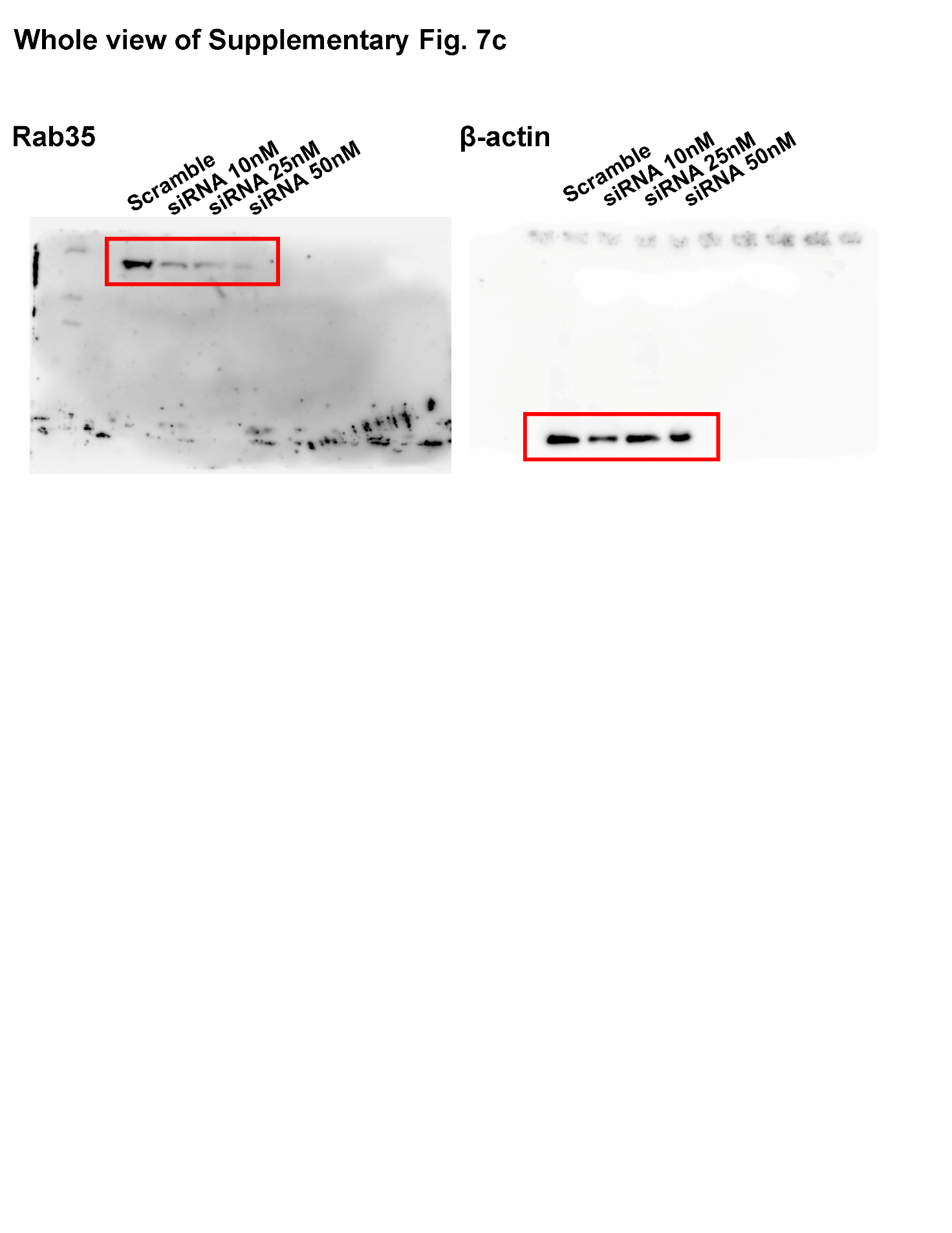
**

**Legends for Supplementary Video files**

**Supplemental Movie 1. Live imaging of vesicle budding in STC-1 cells after stimulation with αSyn** **fibrils (PFFs).** Cells were stained with FM1-43, stimulated with PFFs and z-stacks were collected in intervals of 30s. Cells were imaged for 5,500s.

**Supplemental Movie 2. Live imaging of vesicle budding in SH-SY5Y cells after stimulation with αSyn** **fibrils (PFFs).** Cells were stained with FM1-43, stimulated with PFFs and z-stacks were collected in intervals of 30s. Cells were imaged for 5,500s.
